# Supplementary material for: 2D vanadium carbide MXenzyme to alleviate ROS-mediated inflammatory and neurodegenerative diseases
Source: Nat Commun. 2021 Apr 13;12:2203. doi: 10.1038/s41467-021-22278-x (PMC8044242; doi:10.1038/s41467-021-22278-x)
Supplement: Supplementary file 1 — Supplementary Information [file 41467_2021_22278_MOESM1_ESM.pdf]

Supplementary Information

2D vanadium carbide MXenzyme to alleviate ROS-mediated inflammatory and neurodegenerative diseases

Feng *et al.*

Supplementary Table

Supplementary Table 1 | EDX data of the bulk V_2AlC ceramic. (k ratio = Ratio of characteristic intensities measured on the specimen and standard; Series = Characteristic X-ray lines; Wt% = Concentration in weight percent of the element; Wt% Sigma = Error in the weight percent concentration at the 1 sigma level; Atomic % = Atomic weight percent)

Element	Line Type	Apparent Concentration	k Ratio	Wt%	Wt% Sigma	Atomic %
C	K series	37.96	0.37961	75.59	0.07	92.01
Al	K series	3.39	0.02432	3.85	0.02	2.09
V	K series	15.97	0.15966	20.56	0.07	5.90
Total:				100.00		100.00

Supplementary Table 2 | EDX data of the multilayered V_2C MXene.

Element	Line Type	Wt%	Wt% Sigma	Atomic %
C	K series	15.77	0.22	36.92
Al	K series	0.54	0.05	0.56
V	K series	66.12	0.28	36.51
F	K series	17.57	0.21	26.02
Total:		100.00		100.00

Supplementary Table 3 | EDX data of the few layered V_2C MXene.

Element	Line Type	Wt%	Wt% Sigma	Atomic %
C	K series	19.80	0.19	42.73
O	K series	9.56	0.26	15.49
F	K series	6.83	0.14	9.32
V	K series	63.80	0.29	32.46
Total:		100.00		100.00

Supplementary Figures

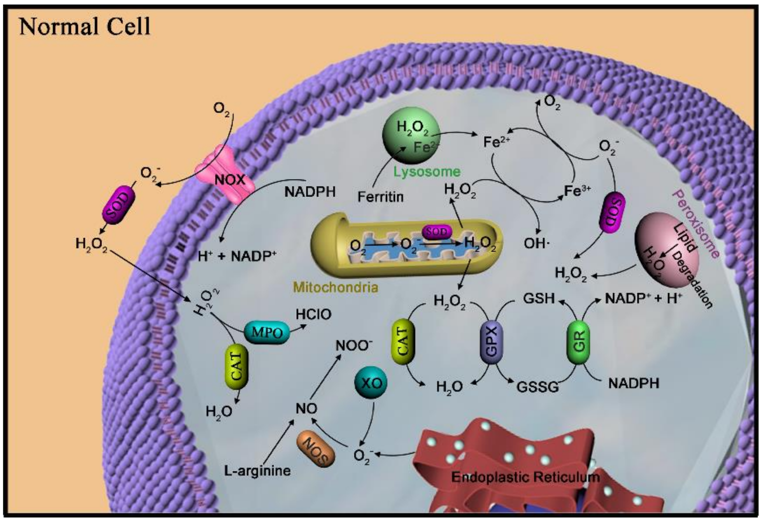

Supplementary Fig. 1 | Schematic illustration of intracellular redox equilibrium in normal cells. In the normal cells, the intracellular redox equilibrium to resist oxidative stress is sustained by a collection of enzymatic antioxidants, primarily consisting of SOD, CAT, POD and GPx.

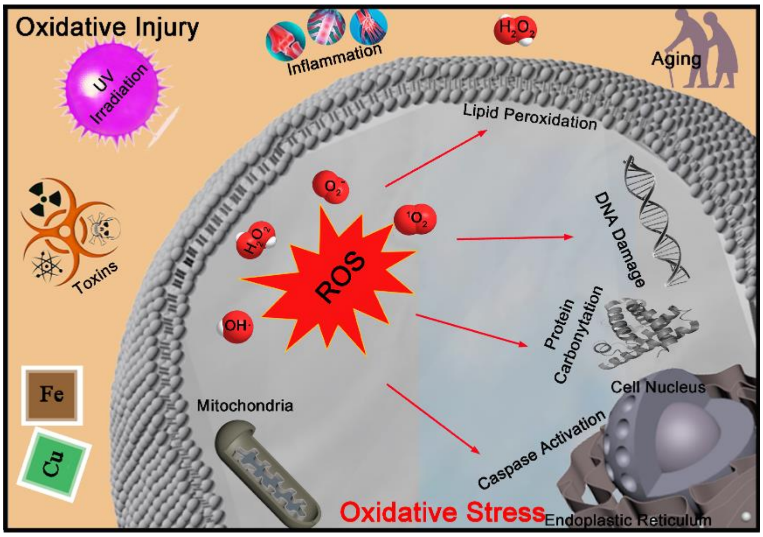

Supplementary Fig. 2 | Schematic illustration of irreversible oxidative damage. Overproduction of ROS is highly deleterious to biosystem, which results in irreversible oxidative damage to the biomacromolecules (e.g., protein, DNA and lipid), induces a variety of cellular responses (e.g., apoptosis and necrosis) and finally involves in pathologies and progression of many diseases, including atherosclerosis, neurodegeneration, inflammation, hemochromatosis and even cancer.

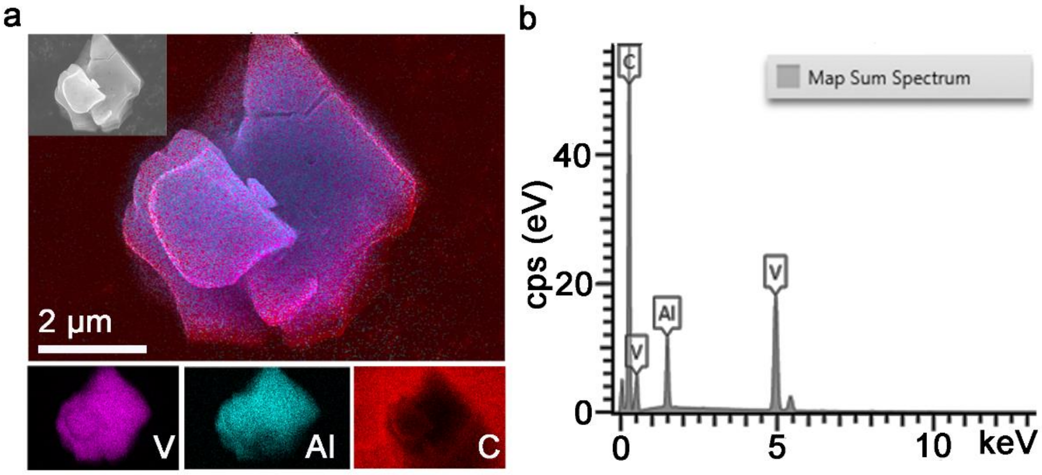

Supplementary Fig. 3 | SEM image and the corresponding element analysis. (**a**) SEM and corresponding elemental-mapping images (V, Al and C elements) of bulk V_2AlC ceramic. Upper inset shows SEM micrograph of V_2AlC. (**b**) EDX spectroscopy of V_2AlC MAX phase powders. A representative image of three replicates from each group is shown in (**a**).

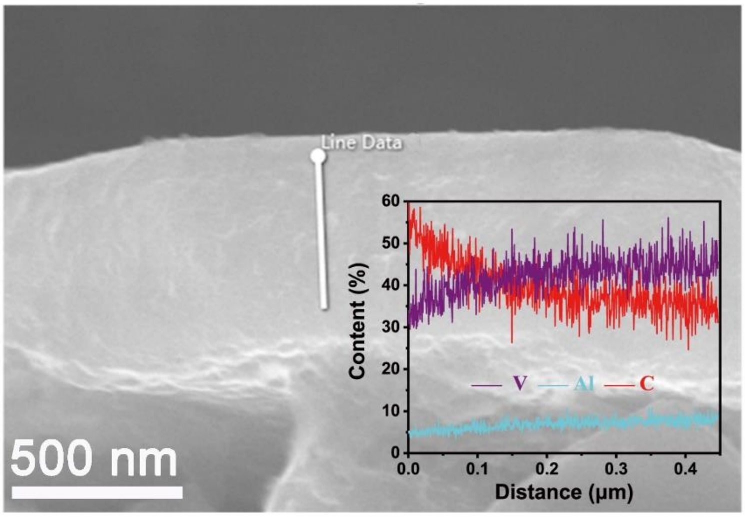

Supplementary Fig. 4 | SEM image of V_2AlC MAX phase powders and EDX linear-scanning profiles of V, Al and C elements along the white line.

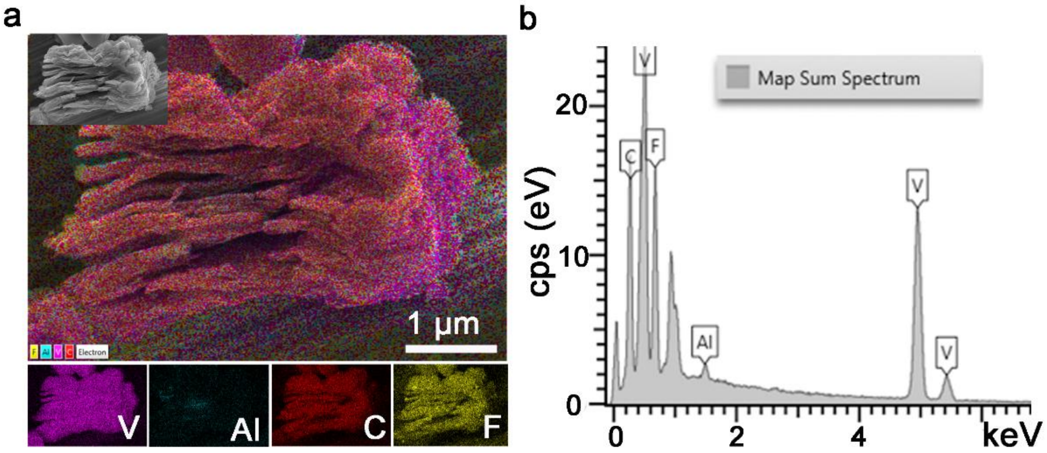

Supplementary Fig. 5 | SEM image and the corresponding element analysis. (**a**) SEM and corresponding elemental-mapping images (V, Al and C elements) of multilayered V_2C MXene. Upper inset shows SEM micrograph of multilayered V_2C MXene. (**b**) EDX spectroscopy of multilayered V_2C MXene. A representative image of three replicates from each group is shown in (**a**).

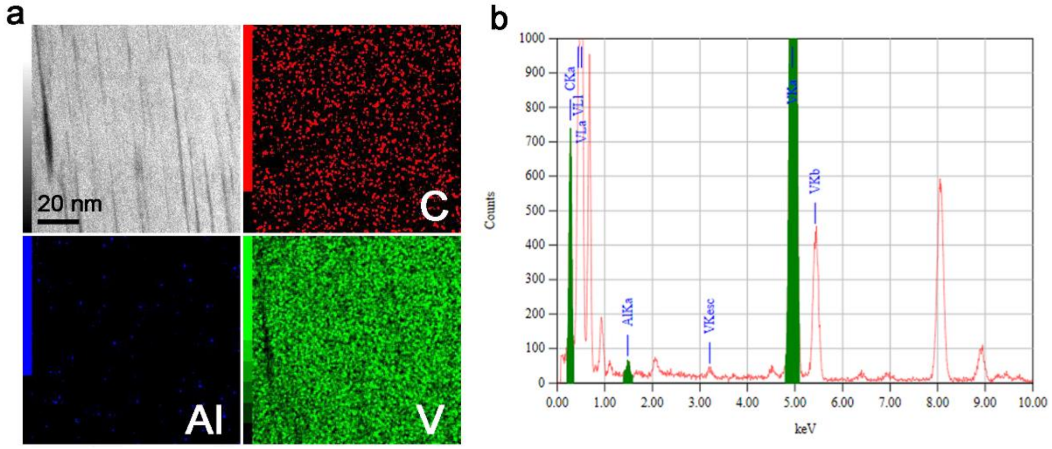

Supplementary Fig. 6 | TEM image and the corresponding element analysis. (**a**) TEM and corresponding elemental-mapping images (V, Al and C elements) of multilayered V_2C MXene. (**b**) EDX spectroscopy of multilayered V_2C MXene. A representative image of two replicates from each group is shown in (**a**).

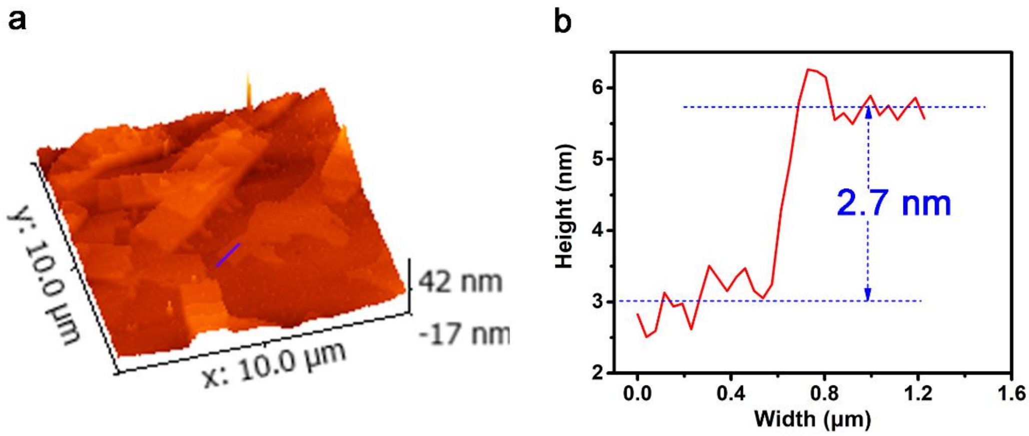

Supplementary Fig. 7 | AFM measurement. (**a**) AFM image of V_2C MXenzyme on cleaved mica. (**b**) Height profile corresponding to the blue line.

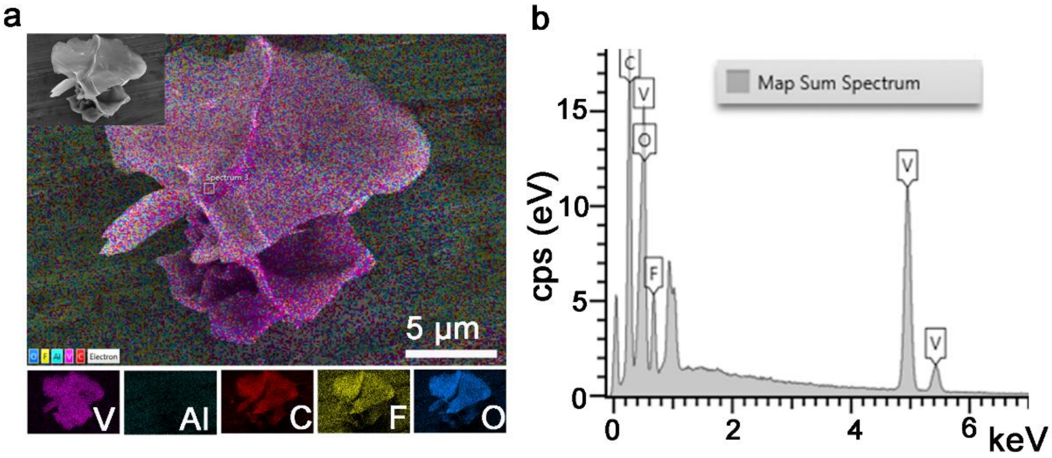

Supplementary Fig. 8 | SEM image and the corresponding element analysis. (**a**) SEM and corresponding elemental-mapping images (V, Al and C elements) of few layered V_2C MXene. Upper inset shows SEM micrograph of few layered V_2C MXene. (**b**) EDX spectroscopy of few layered V_2C MXene. A representative image of three replicates from each group is shown in (**a**).

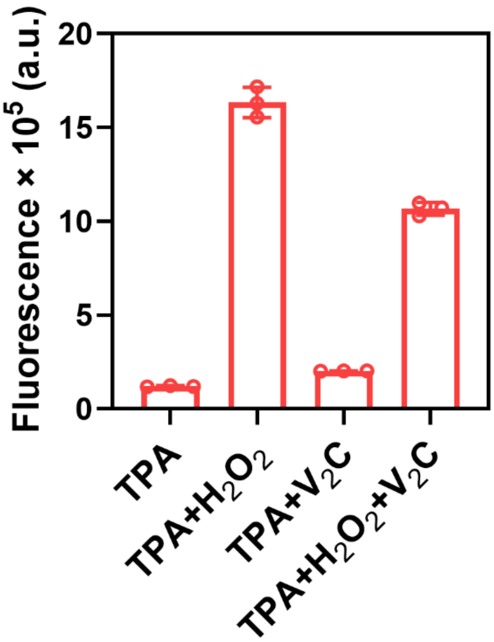

Supplementary Fig. 9 | Corresponding fluorescence values of TPA after different treatments (n = 3 for each group, data presented as Mean ± SD).

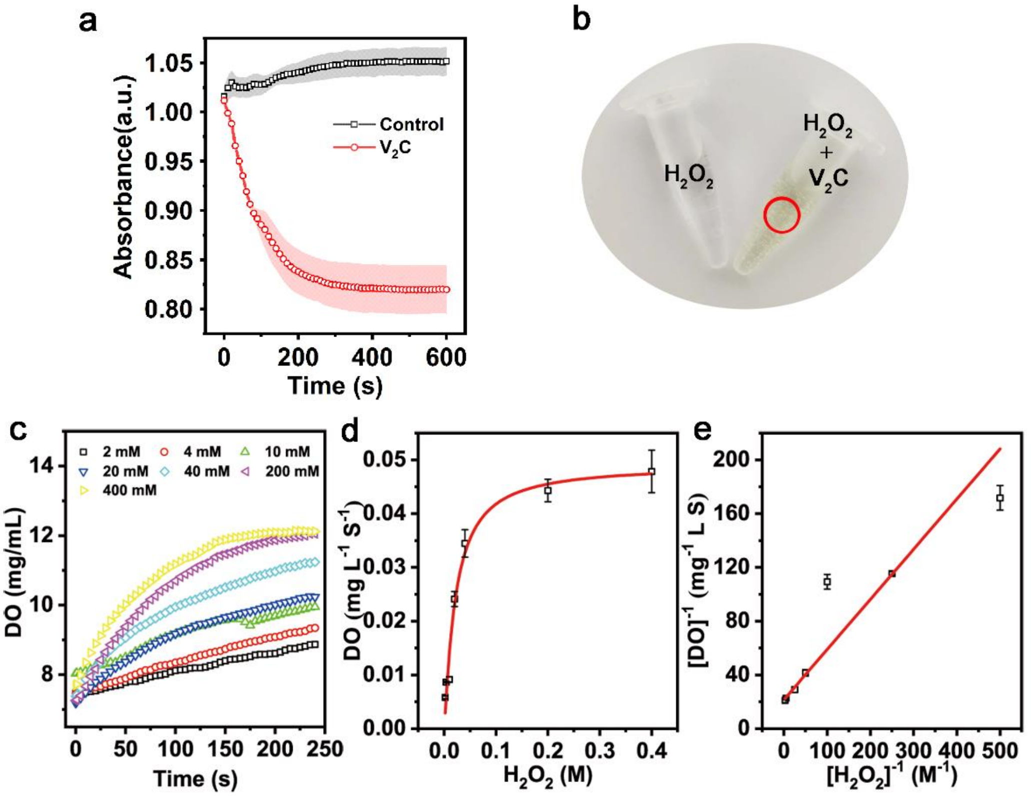

Supplementary Fig. 10 | CAT-like activity of V_2C MXenzyme. (**a**) Time-dependent absorbance changes at 240 nm in the presence of H_2O_2 with or without V_2C MXenzyme (n = 3 for each group, data presented as Mean ± SD). (**b**) Formation of bubbles indicating the decomposition of H_2O_2 into O_2 by the V_2C MXenzyme. (**c**) Time-dependent O_2 generation in the presence of V_2C MXenzyme at various H_2O_2 concentrations. (**d**) Michaelis-Menten curve and (**e**) Lineweaver-Burk plots of the CAT-like activity of V_2C MXenzyme. The K_m value of the V_2C MXenzyme was 9.16 mM, and the V_{max} value was 30 mM/s (n = 3 for each group, data presented as Mean ± SD).

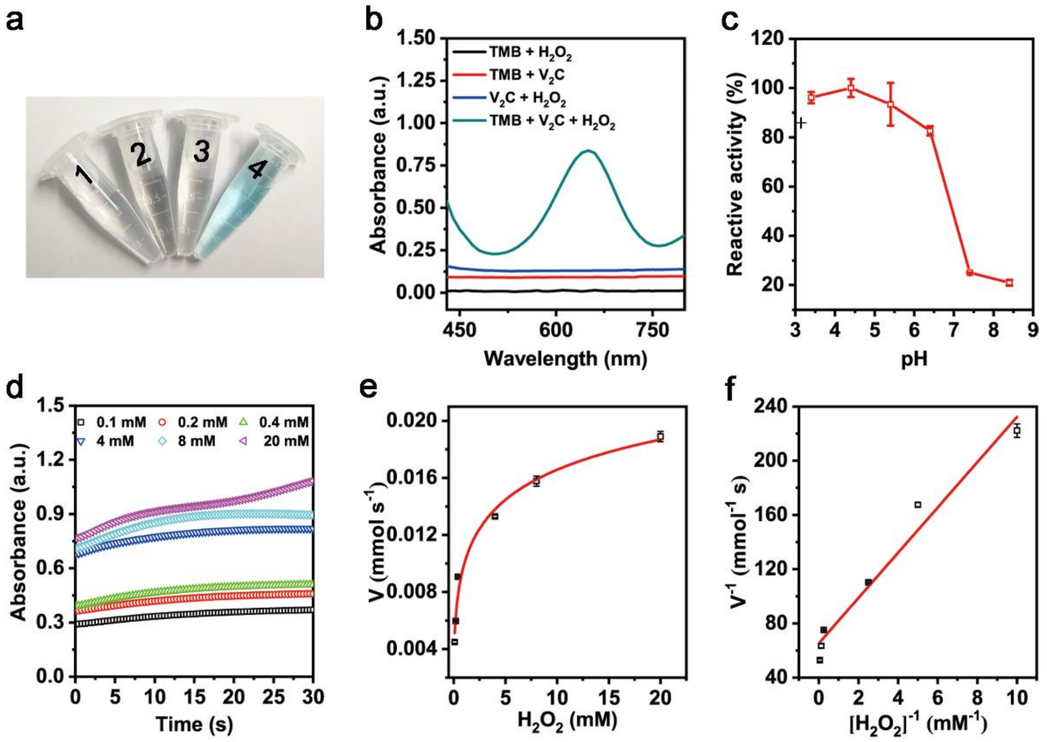

Supplementary Fig. 11 | POD-like activity of V_2C MXenzyme. (**a**) Photography of the mixture of (1) TMB + H_2O_2, (2) TMB + V_2C, (3) V_2C + H_2O_2 and (4) TMB + V_2C + H_2O_2. (**b**) UV-Vis absorption spectra measured in different reaction systems: (1) TMB + H_2O_2, (2) TMB + V_2C, (3) V_2C + H_2O_2 and (4) TMB + V_2C + H_2O_2. (**c**) pH-dependent POD-like activity of V_2C MXene (n = 3 for each group, data presented as Mean ± SD). (**d**) Time-dependent absorbance changes of TMB in presence of V_2C MXene at various H_2O_2 concentrations. (**e**) Michaelis-Menten curve and (**f**) Lineweaver-Burk plots of POD-like activity of V_2C MXenzyme. The K_m value of the V_2C MXenzyme was 0.26 mM, and the V_{max} value was 0.015 mM/s. (n = 3 for each group, data presented as Mean ± SD).

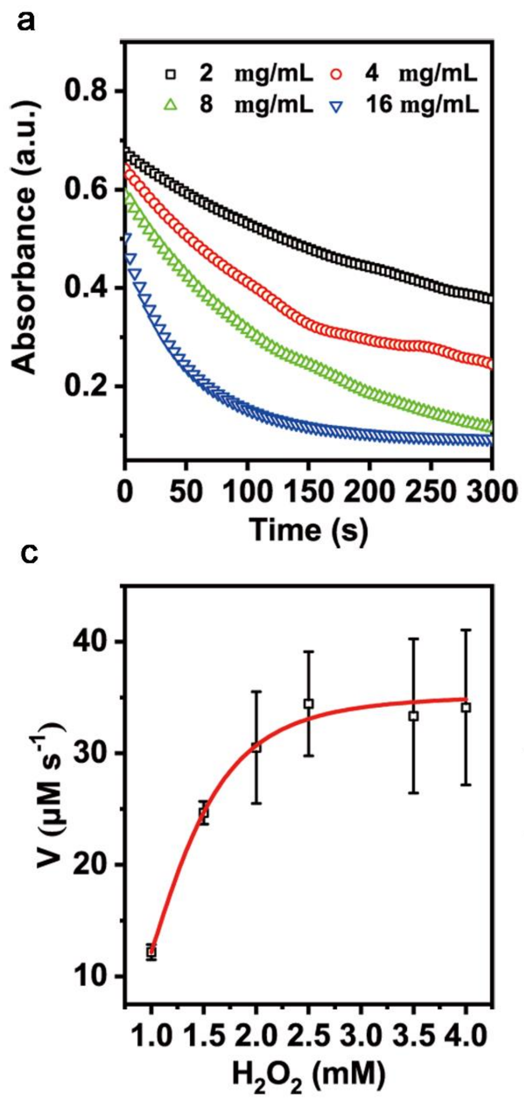

Supplementary Fig. 12 | GPx-like activity of V_2C MXenzyme. (**a**) Time-dependent absorbance changes of NADPH in presence of different concentrations of V_2C MXenzyme. (**b**) Time-dependent absorbance changes of NADPH at various H_2O_2 concentrations. (**c**) Michaelis-Menten curve and (**d**) Lineweaver-Burk plots of GPx-like activity of the V_2C MXenzyme (n = 3 for each group, data presented as Mean ± SD). The K_m value of the V_2C MXenzyme was 10.86 mM, and the V_{max} value was 0.16 mM/s.

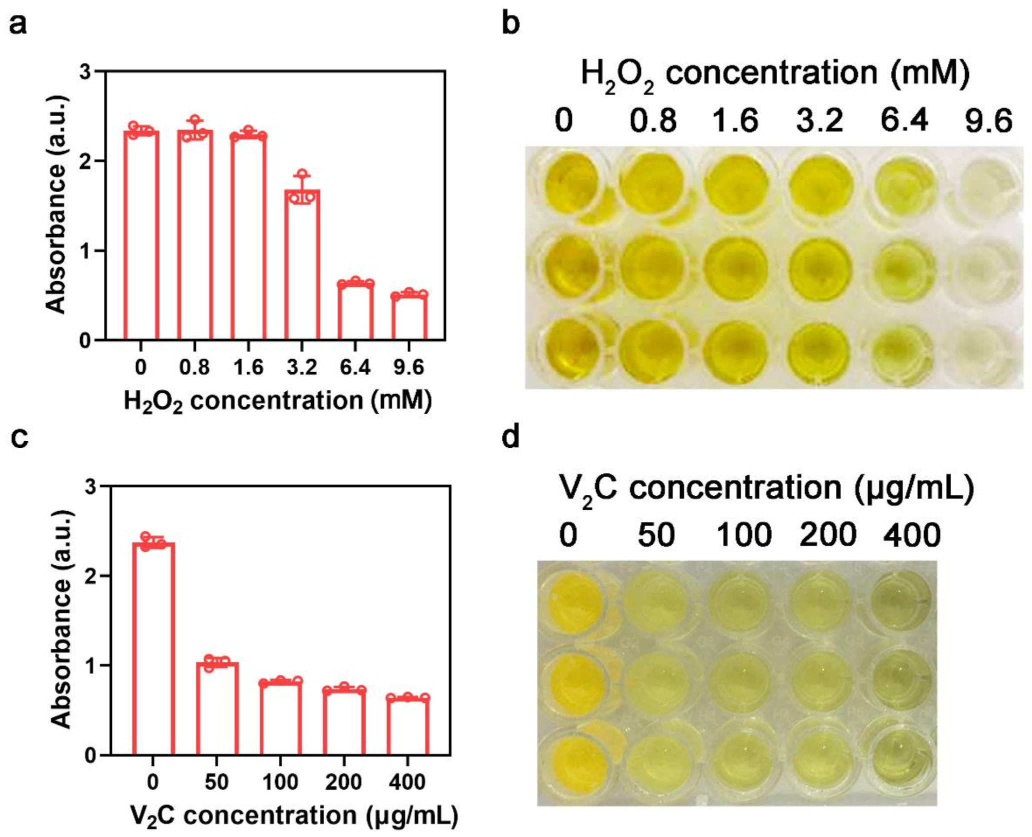

Supplementary Fig. 13 | TPx-like activity of V_2C MXenzyme. (**a**) H_2O_2 concentration-dependent absorbance changes (n = 3 for each group, data presented as Mean ± SD) and (**b**) corresponding photograph of DTNB in the presence of V_2C MXenzyme and cysteine at various H_2O_2 concentrations. (**c**) V_2C MXene concentration-dependent absorbance changes (n = 3 for each group, data presented as Mean ± SD) and (**d**) corresponding photograph of DTNB in the presence of H_2O_2 and cysteine at various concentrations of V_2C MXene.

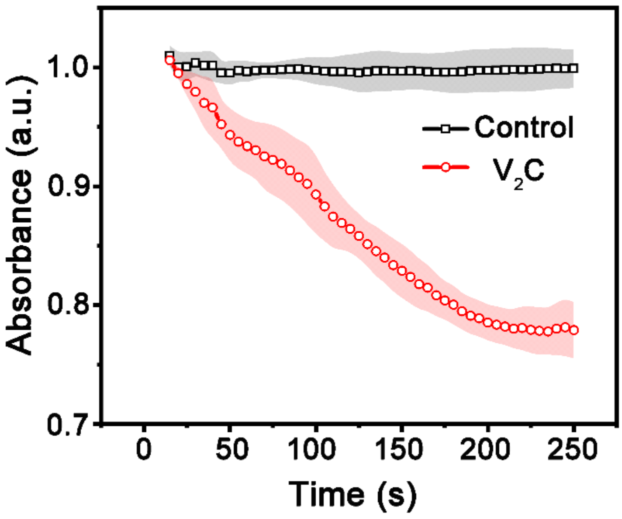

Supplementary Fig. 14 | HPO-like activity of V_2C MXenzyme. The absorbance changes of the reaction mixture (10 μM H_2O_2, 1 mM Br^-, 50 μM MCD, 50 μg/mL V_2C MXene) at 290 nm as measured in time-scanning mode (n = 3 for each group, data presented as Mean ± SD).

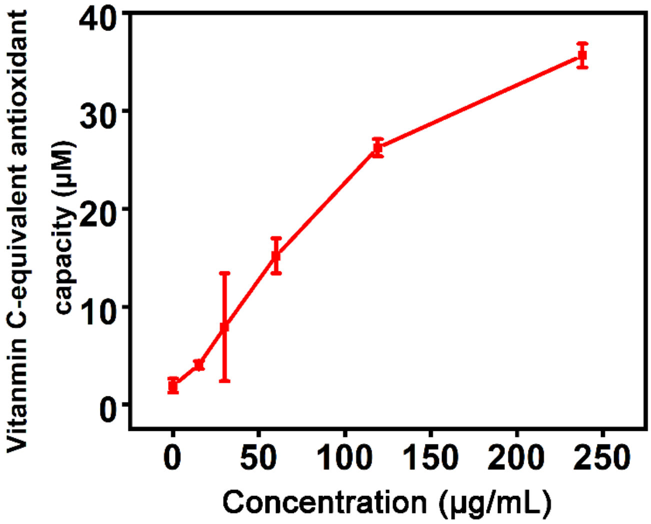

Supplementary Fig. 15 | Total antioxidant capacity of V_2C MXenzyme compared with Vitamin C (n = 3 for each group, data presented as Mean ± SD)**.**

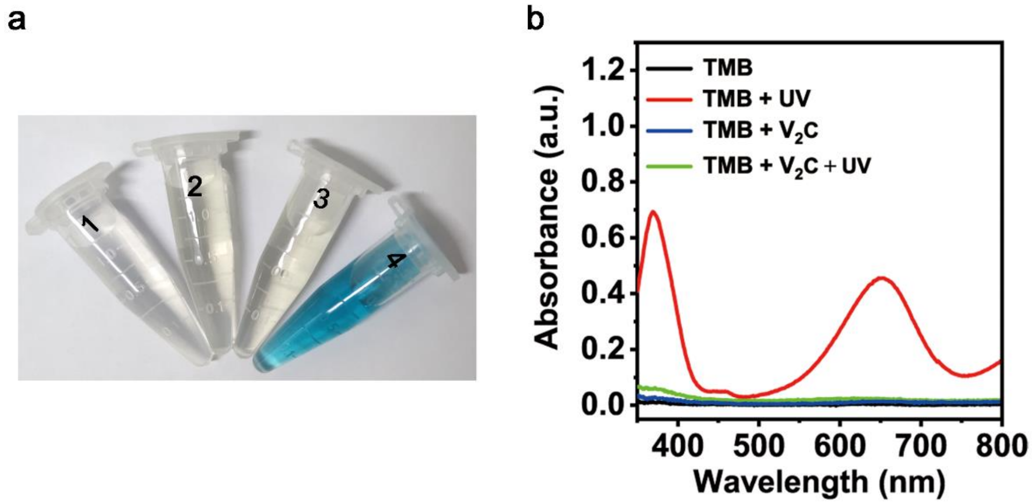

Supplementary Fig. 16 | UV inhibition activity of V_2C MXenzyme. (**a**) Photography of the mixture of (1) TMB, (2) TMB + V_2C, (3) TMB + V_2C + UV, and (4) TMB + UV. (**b**) UV-Vis absorption spectra measured in different reaction systems: (1) TMB, (2) TMB + UV, (3) TMB + V_2C, and (4) TMB + V_2C + UV.

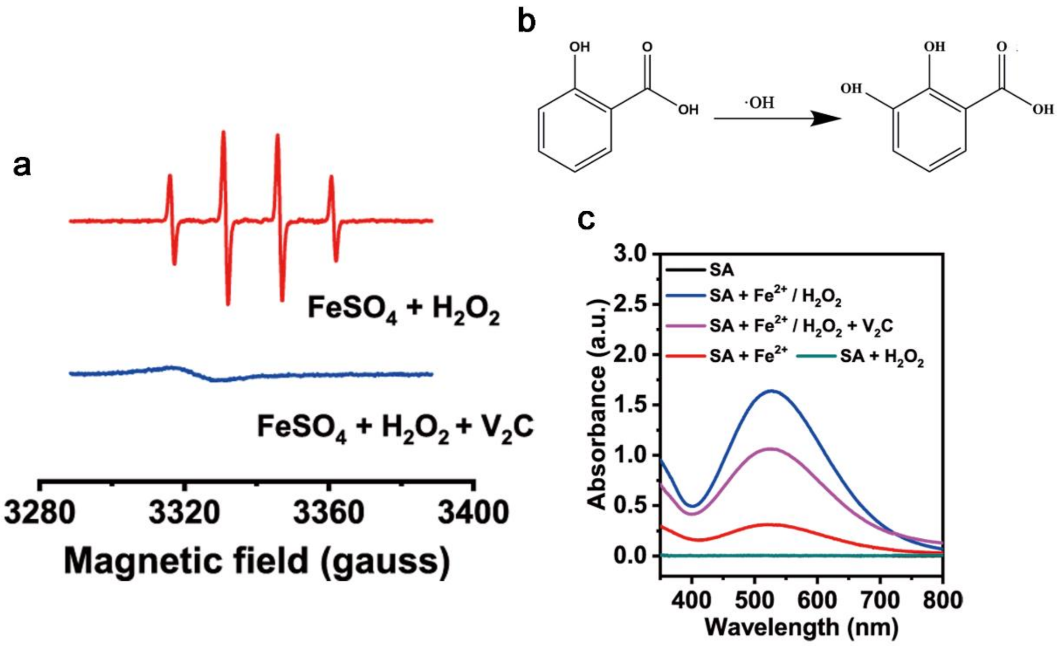

Supplementary Fig. 17 | •OH-scavenging activity of V_2C MXenzyme. (**a**) ESR spectra of the effect of V_2C MXenzyme on •OH scavenging. (**b**) Chemical formulas of the reaction between salicylic acid (SA) and Fenton reagent. (**c**) Absorption spectra of SA after different treatments.

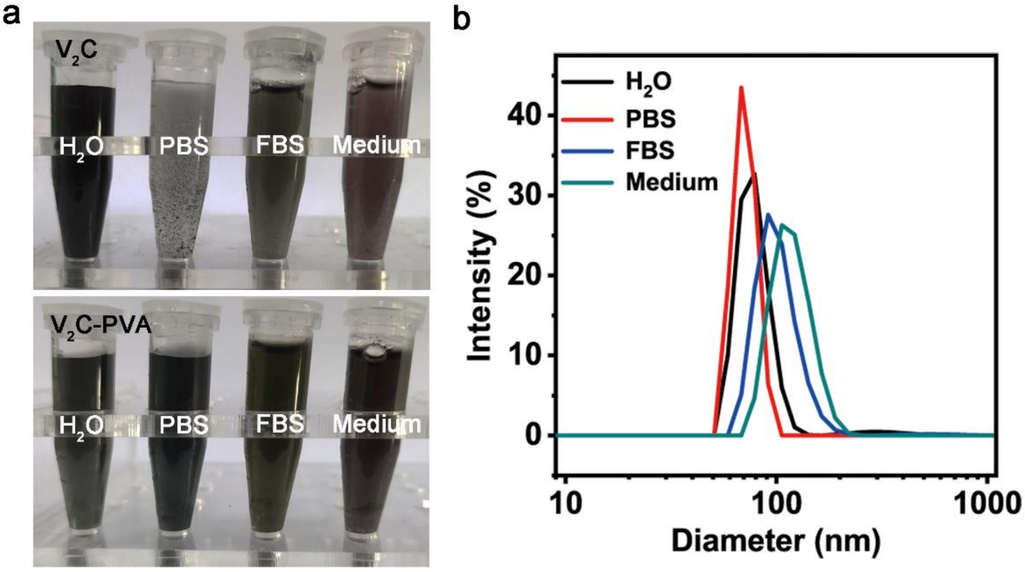

Supplementary Fig. 18 | Colloidal stability of PVA-modified V_2C MXenzyme. (**a**) Photographic images of V_2C MXene with or without PVA modification in various physiological solutions including water, PBS, FBS and cell culture medium. (**b**) Hydrodynamic diameters of V_2C MXenzyme after PVA modification in various physiological solutions including water, PBS, FBS and cell culture medium.

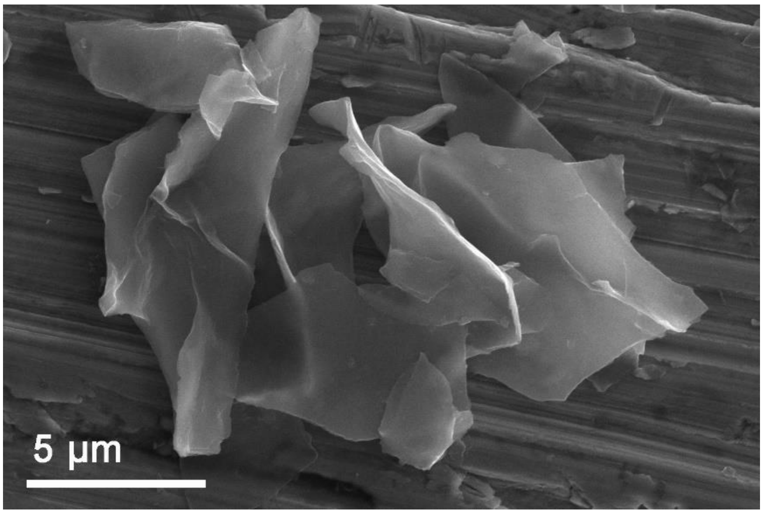

Supplementary Fig. 19 | SEM image of PVA-modified V_2C MXenzyme. A representative image of three replicates from each group is shown.

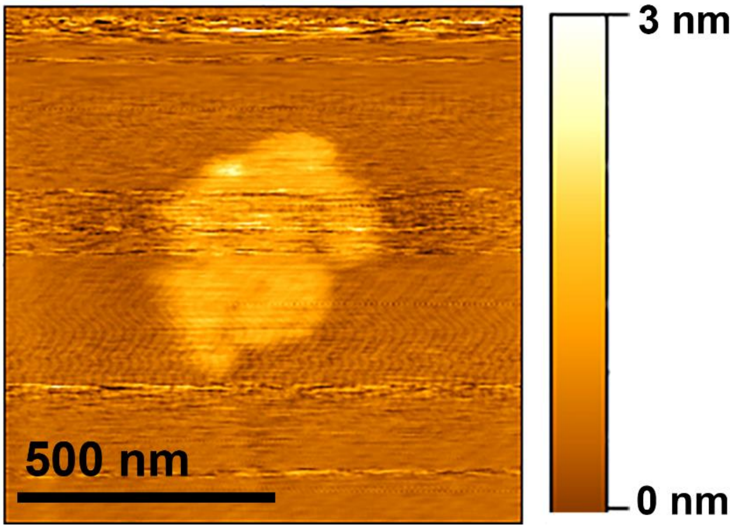

Supplementary Fig. 20 | AFM image of PVA-modified V_2C MXenzyme.

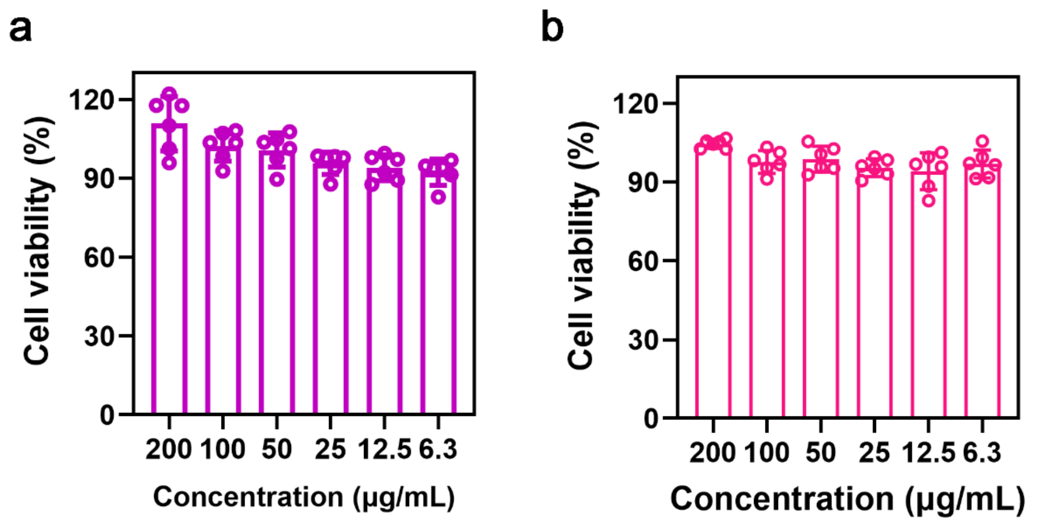

Supplementary Fig. 21 | Cytotoxicity of V_2C MXenzyme. Cell viabilities of V_2C MXenzyme as determined in (**a**) L929 cells and (**b**) PC 12 cells using CCK-8 assay at elevated concentrations (n = 6 for each group, data presented as Mean ± SD).

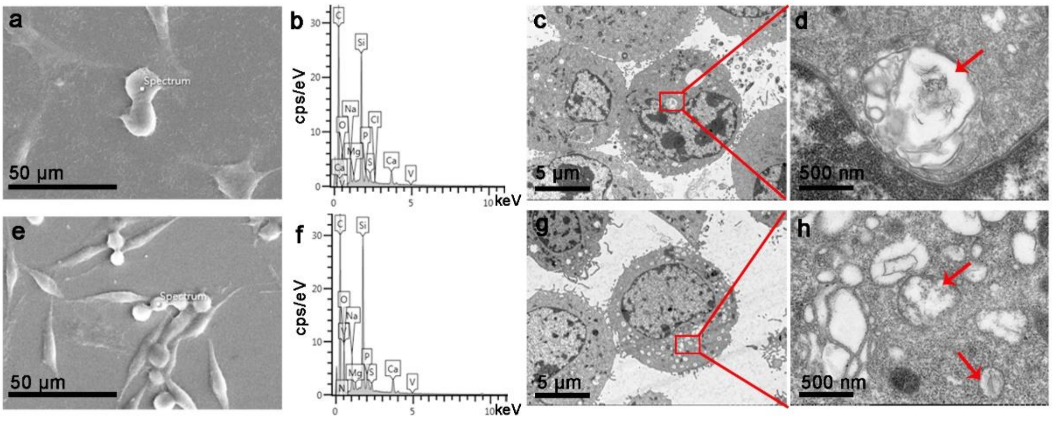

Supplementary Fig. 22 | Cellular uptake of V_2C MXenzyme. (**a**) SEM image, (**b**) corresponding EDX spectrum, (**c**) TEM image and (**d**) magnified TEM image showing the internalization of V_2C MXenzyme in L929 cells after 24 h uptake. (**e**) SEM image, (**f**) corresponding EDX spectrum, (**g**) TEM image and (**h**) magnified TEM image showing internalization of V_2C MXenzyme in PC12 cells after 24 h uptake. A representative image of three biological replicates from each group is shown.

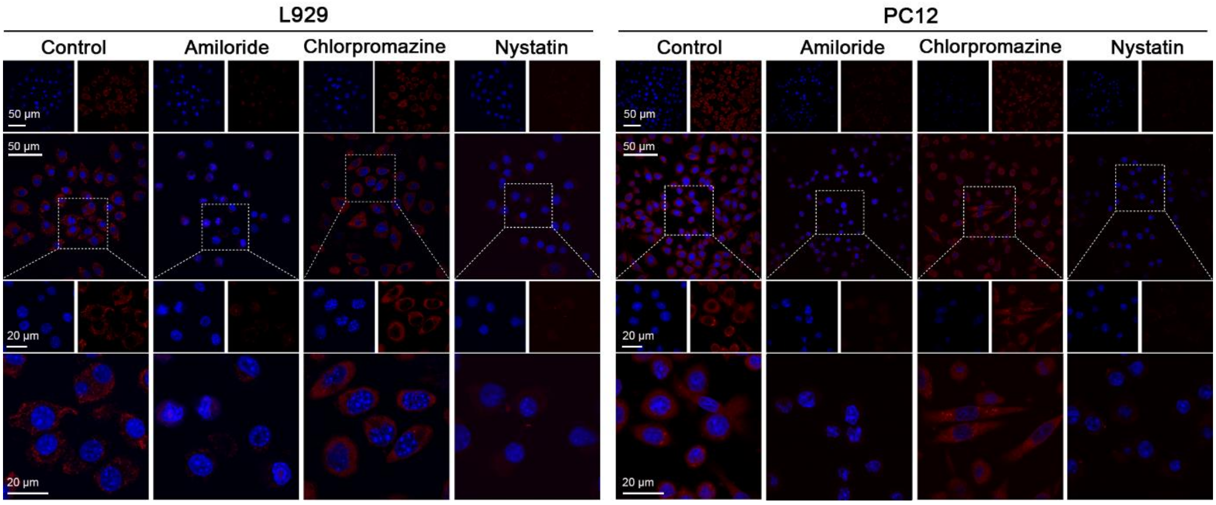

Supplementary Fig. 23 | CLSM images of cellular uptake mechanism of V_2C MXenzyme. L929 cells and PC 12 cells incubated with V_2C MXenzyme for 4h in the absence or presence of inhibitor amiloride, chlorpromazine and nystatin. A representative image of three biological replicates from each group is shown.

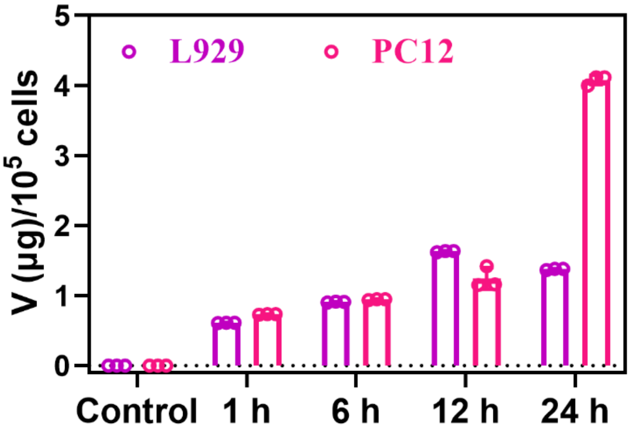

Supplementary Fig. 24 | Internalization of V_2C MXenzyme in both L929 cells and PC12 cells as measured by ICP-OES and represented as amount of V uptake per 1×10^5 cells (n = 3 for each group, data presented as Mean ± SD)**.**

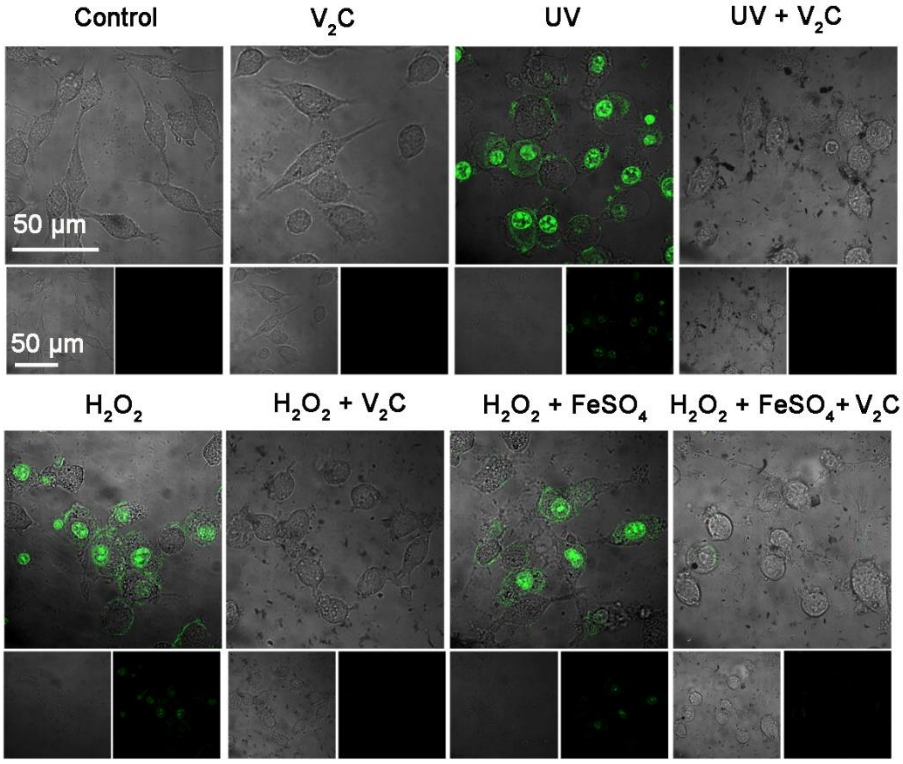

Supplementary Fig. 25 | CLSM images of Caspase-3/7 activity of L929 cells after different treatments. A representative image of three biological replicates from each group is shown.

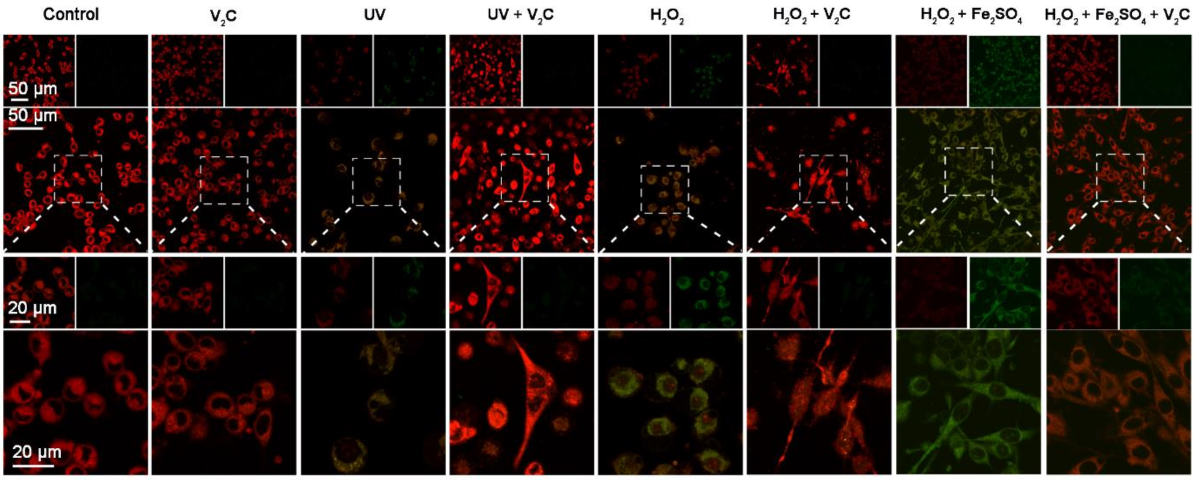

Supplementary Fig. 26 | CLSM images of lipid peroxidation of C11-BODIPY$^{581/591}$-stained PC 12 cells after different treatments. A representative image of three biological replicates from each group is shown.

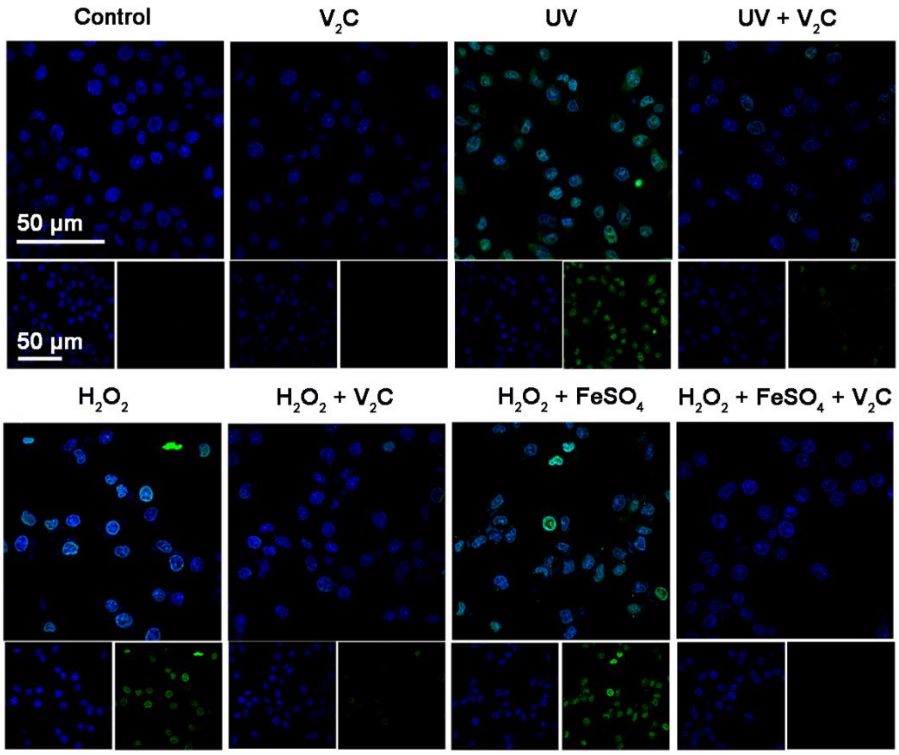

Supplementary Fig. 27 | Representative immunofluorescence CLSM images of γH2AX DNA damage foci in PC12 cells after different treatments. A representative image of three biological replicates from each group is shown.

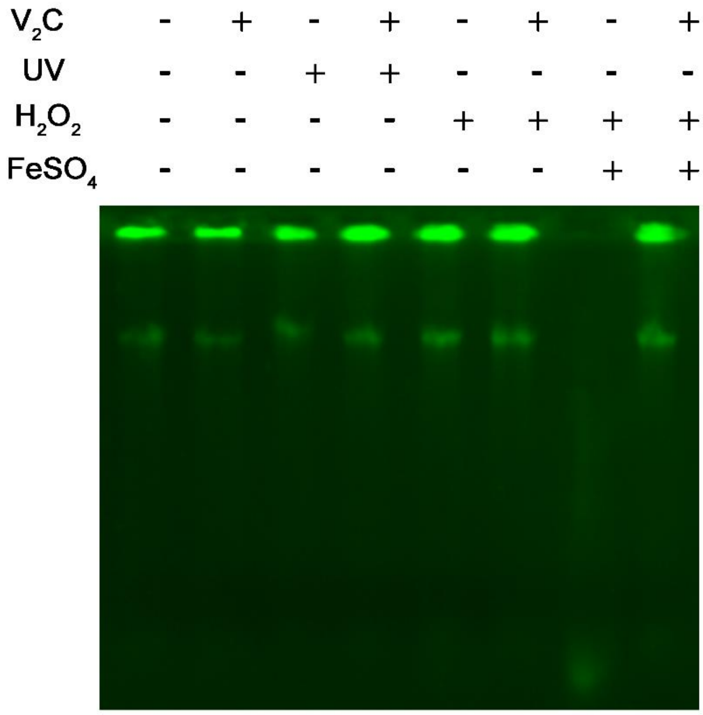

Supplementary Fig. 28 | In vitro DNA protection assay after different treatments.

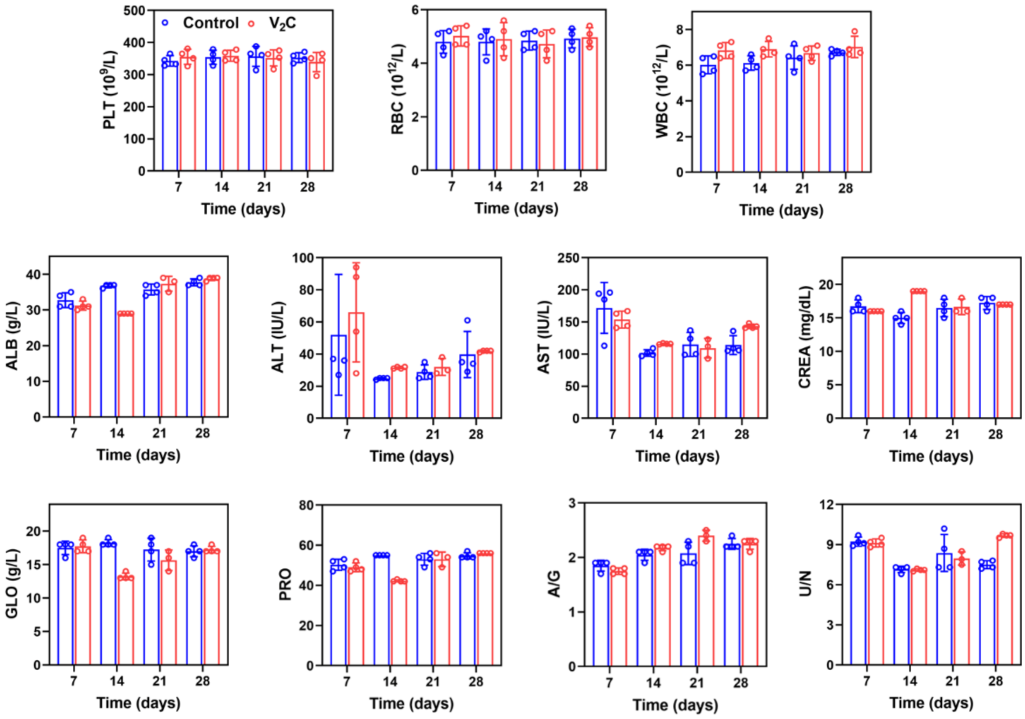

Supplementary Fig. 29 | Hematology and blood biochemical examination of the mice treated with V_2C MXenzyme (15 mg/kg) 7, 14, 21 and 28 days after injection. The indexes include platelets (PLT), red blood cell count (RBC), white blood cell count (WBC), albumin (ALB), alanine aminotransferase (ALT), aspartate transaminase (AST), creatinine (CERA), globulin (GLO), blood protein (PRO), the ratio of albumin to globulin (A/G) and urea nitrogen (UN) (n = 4 for each group, data presented as Mean ± SD).

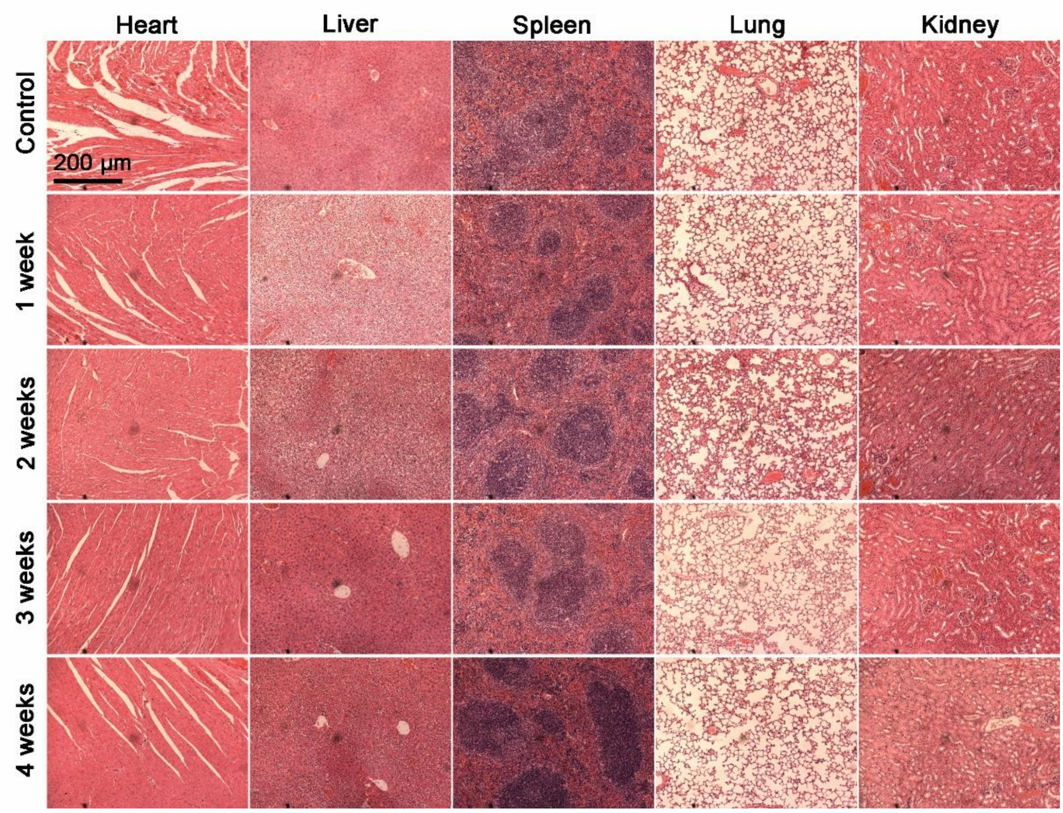

Supplementary Fig. 30 | H&E-stained major organs sections (heart, liver, spleen, lung and kidney) obtained from mice treated with V_2C MXenzyme (15 mg/kg) 1, 2, 3 and 4 weeks post-injection. A representative image of three biological replicates from each group is shown.

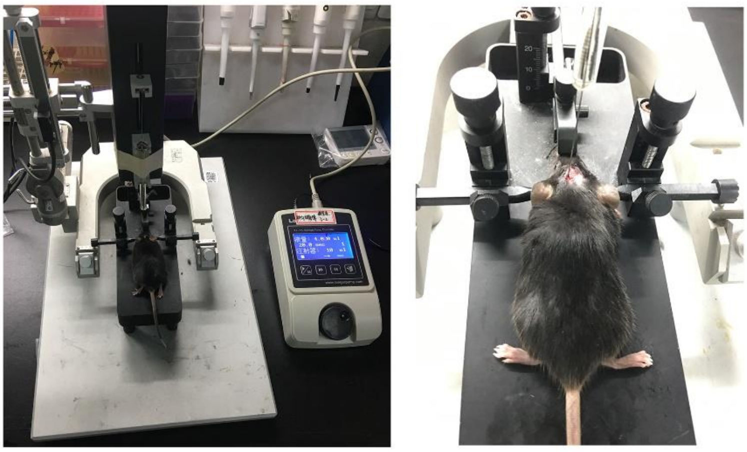

Supplementary Fig. 31 | Pictures for stereotaxic surgery.

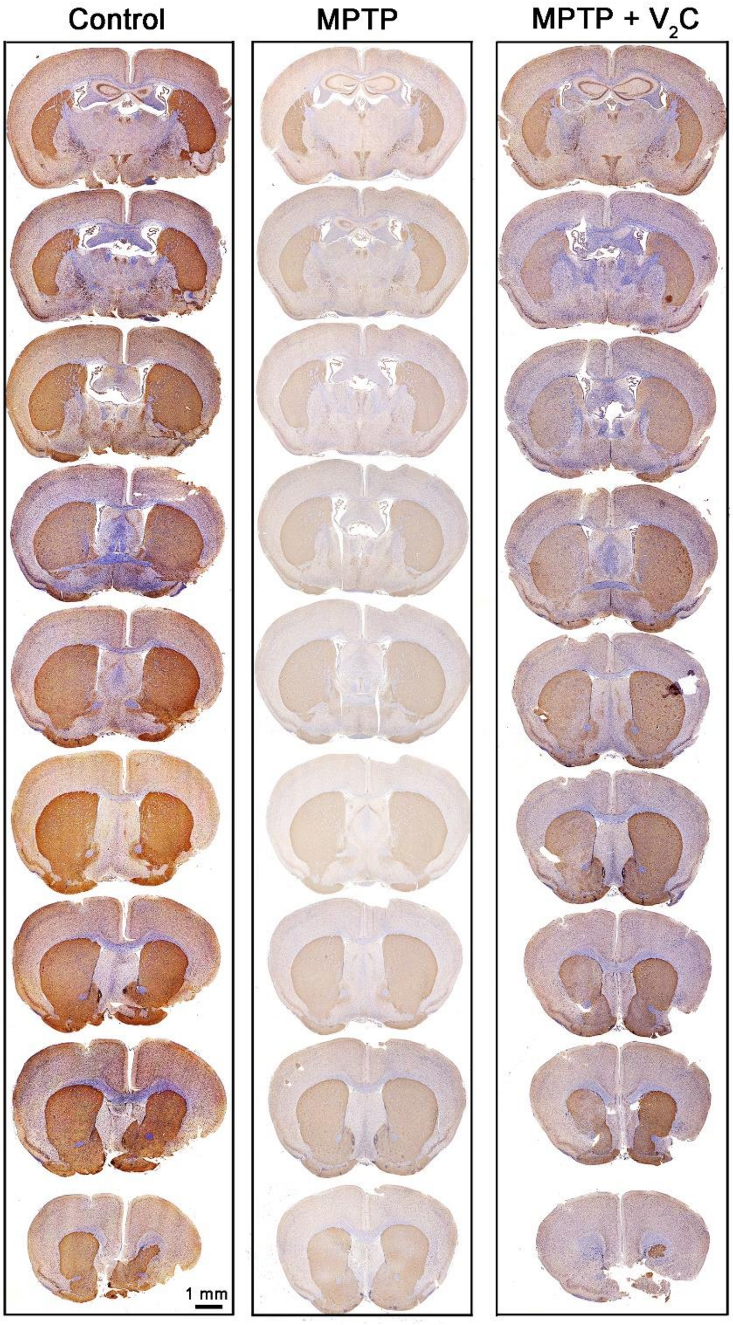

Supplementary Fig. 32 | Immunohistochemistry images of TH expression in the brains of mice after different treatments.

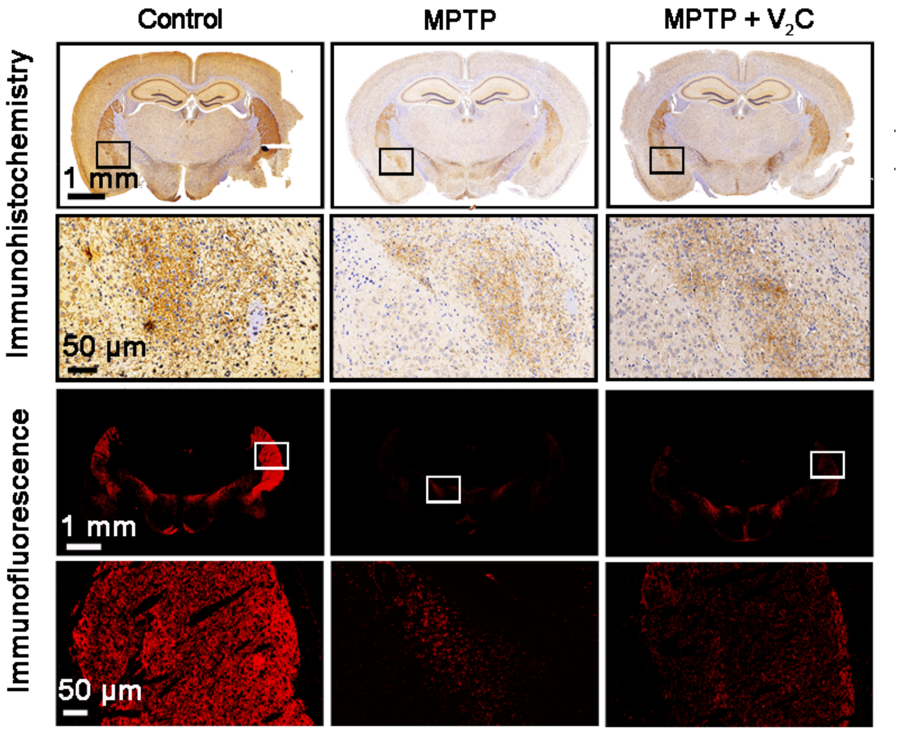

Supplementary Fig. 33 | Immunohistochemistry and immunohistofluorescence images of TH expression in the brains of mice after different treatments (coronal plane, enlarged Fig. 9j). A representative image of three biological replicates from each group is shown.

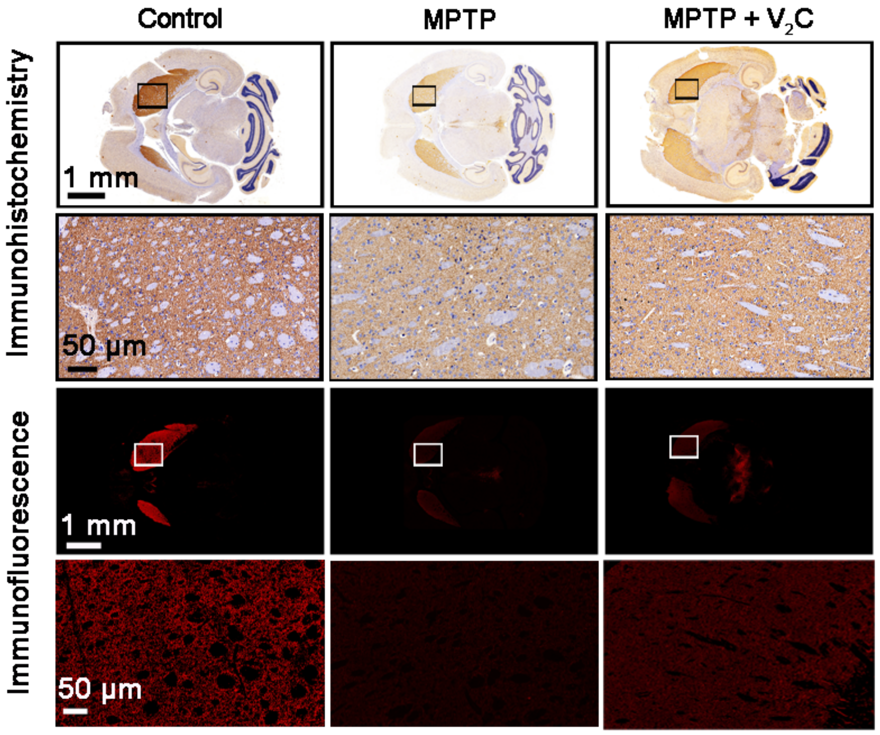

Supplementary Fig. 34 | Immunohistochemistry and immunofluorescence images of TH expression in the brains of mice after different treatments (transverse plane). A representative image of three biological replicates from each group is shown.

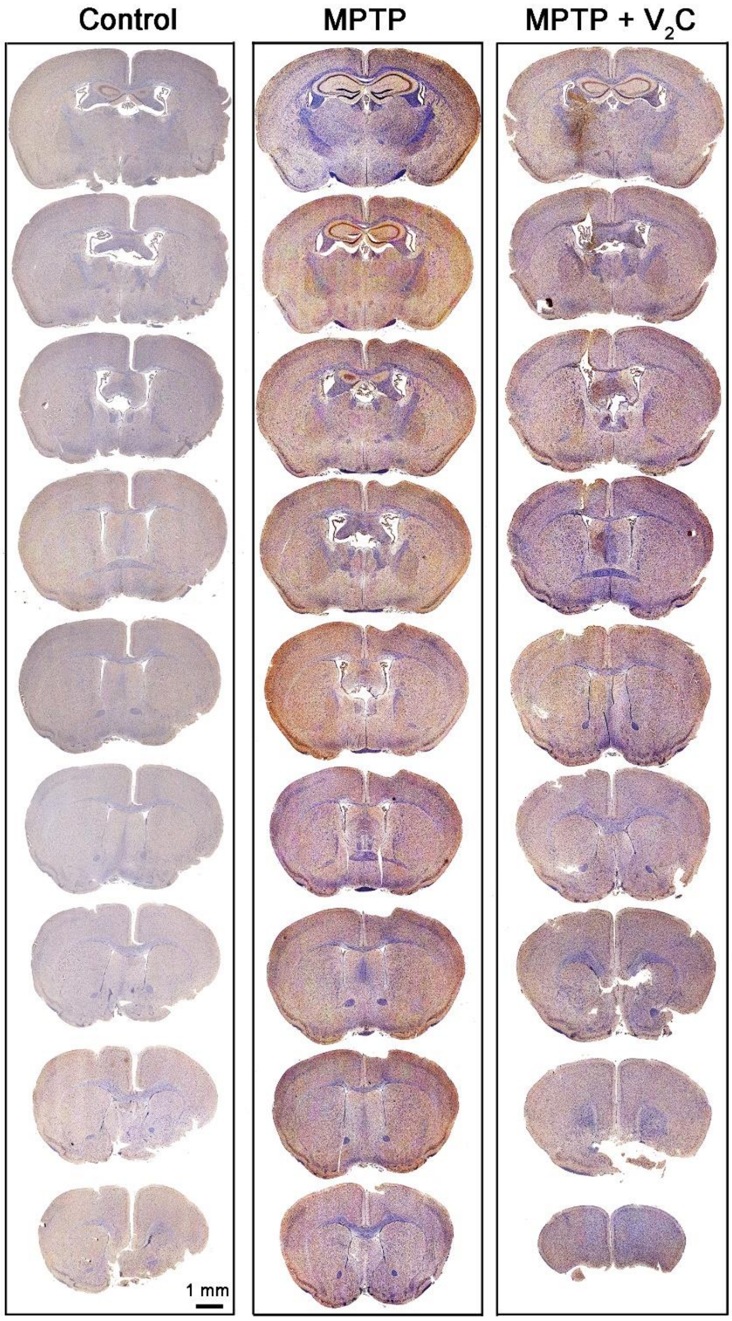

Supplementary Fig. 35 | Immunohistochemistry images of IBA-1 expression in the brains of mice after different treatments.

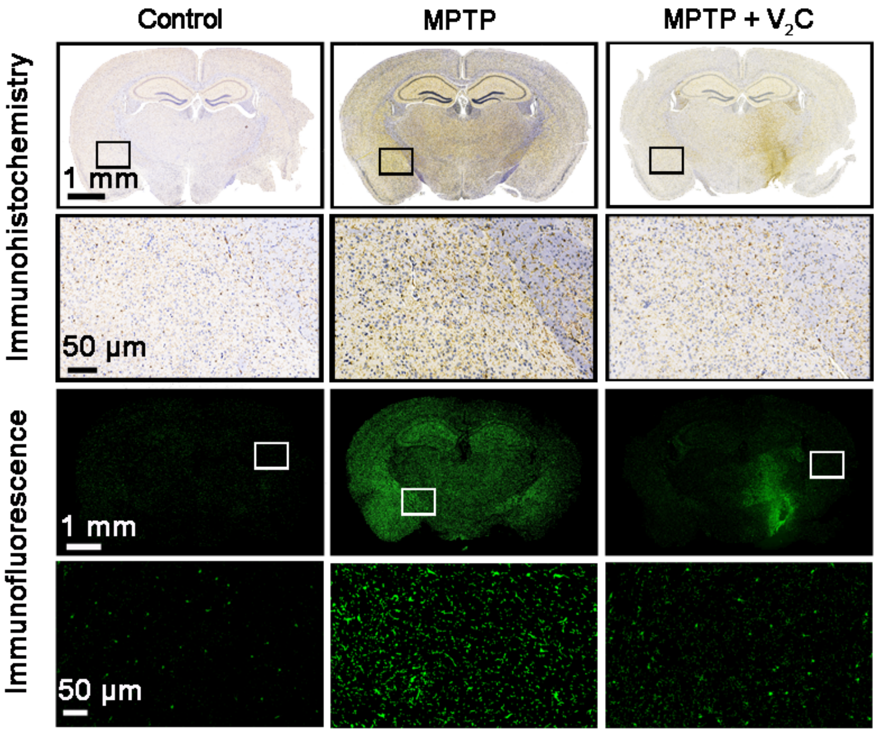

Supplementary Fig. 36 | Immunohistochemistry and immunofluorescence images of IBA-1 expression in the brains of mice after different treatments (coronal plane). A representative image of three biological replicates from each group is shown.

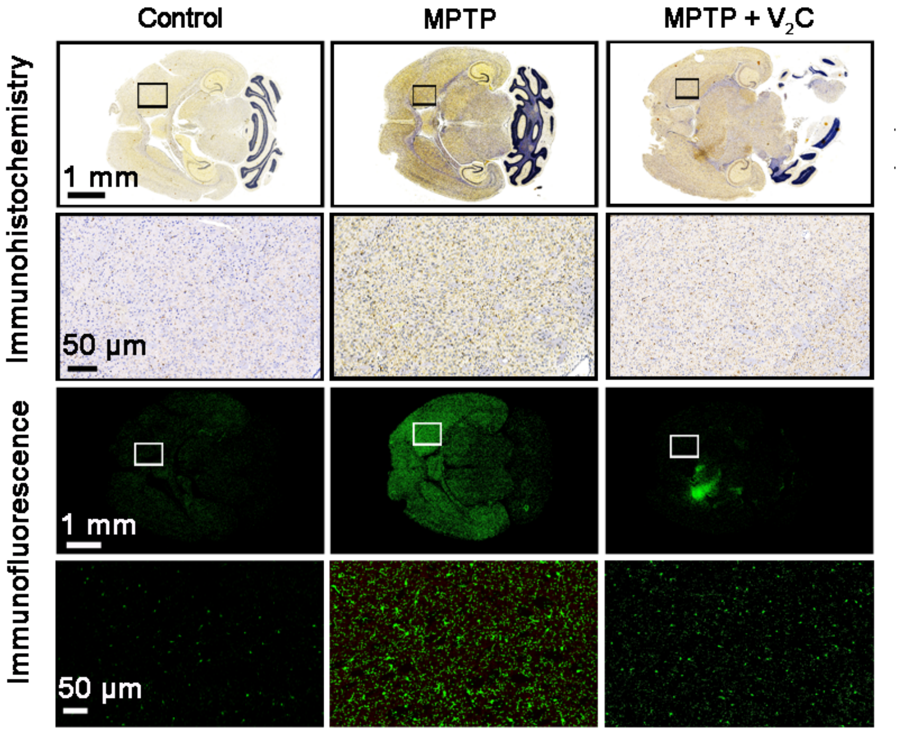

Supplementary Fig. 37 | Immunohistochemistry and immunofluorescence images of IBA-1 expression in the brains of mice after different treatments (transverse plane). A representative image of three biological replicates from each group is shown.

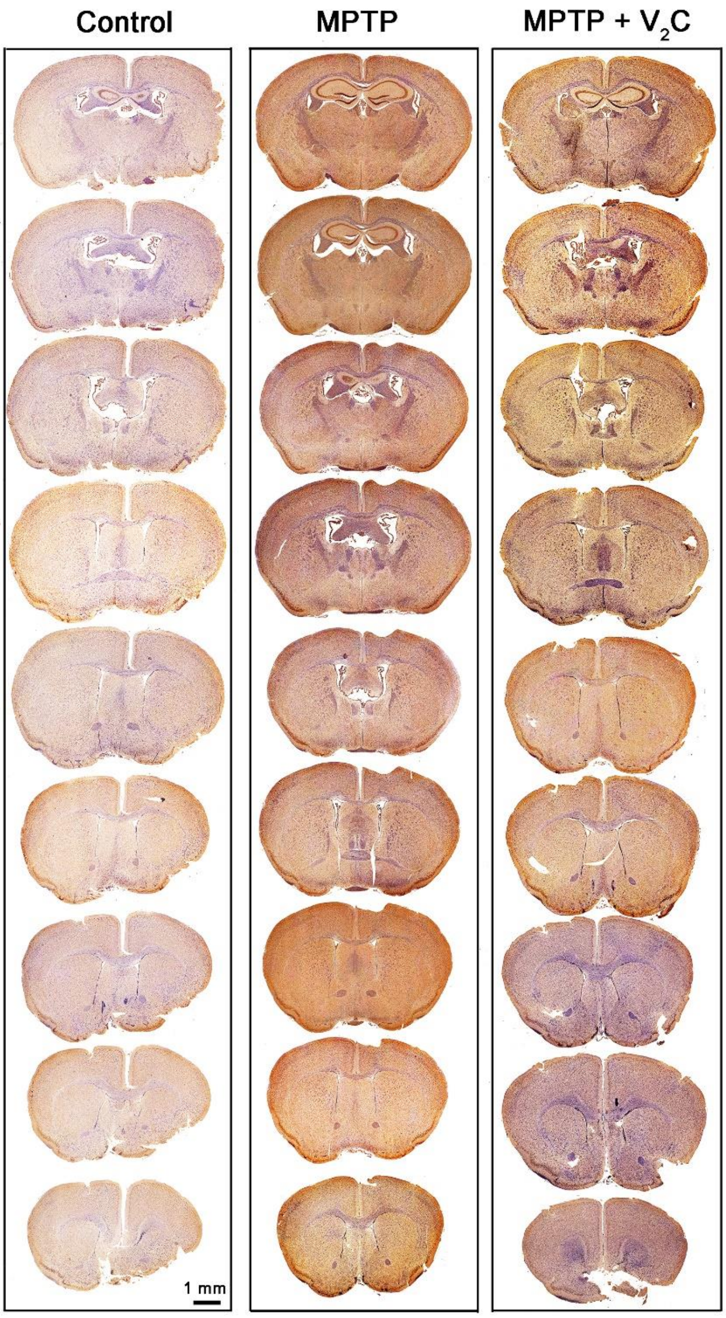

Supplementary Fig. 38 | Immunohistochemistry images of 4-HNE expression in the brains of mice after different treatments.

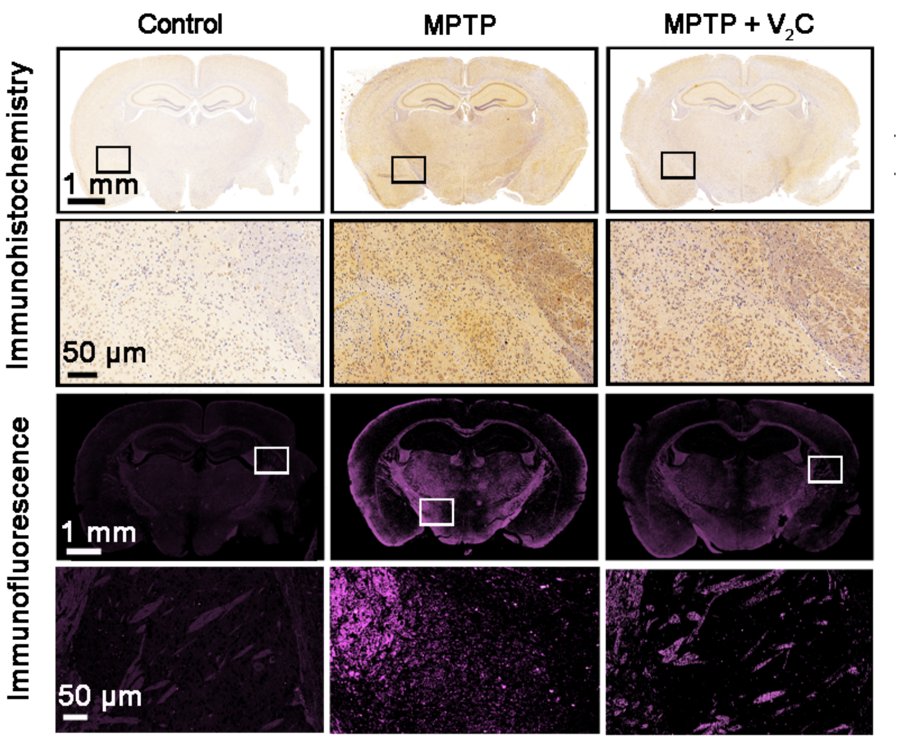

Supplementary Fig. 39 | Immunohistochemistry and immunofluorescence images of 4-HNE expression in the brains of mice after different treatments (coronal plane). A representative image of three biological replicates from each group is shown.

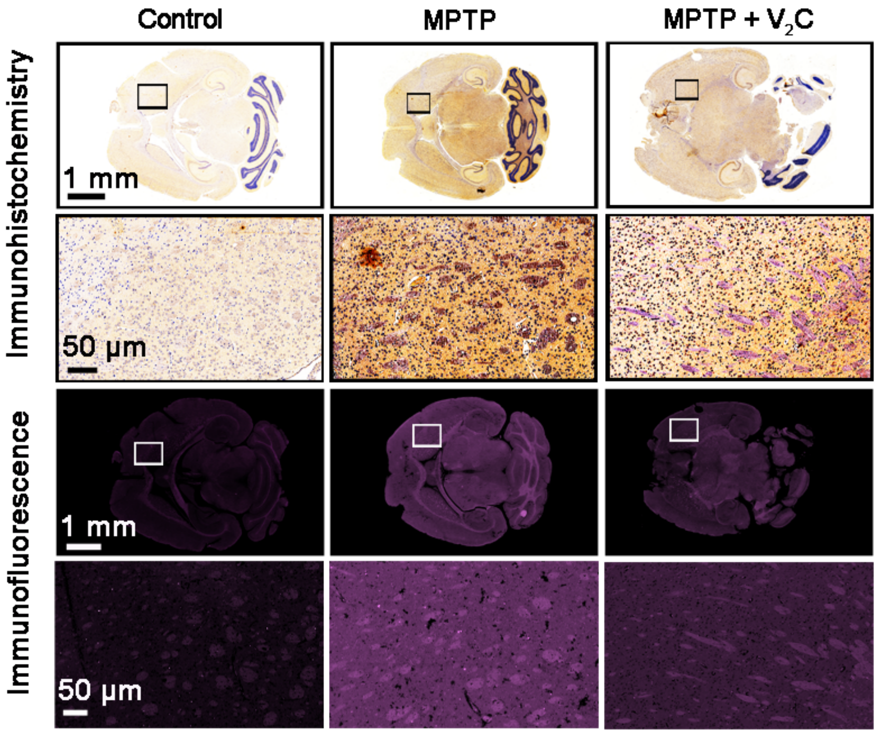

Supplementary Fig. 40 | Immunohistochemistry and immunofluorescence images of 4-HNE expression in the brains of mice after different treatments (transverse plane). A representative image of three biological replicates from each group is shown.

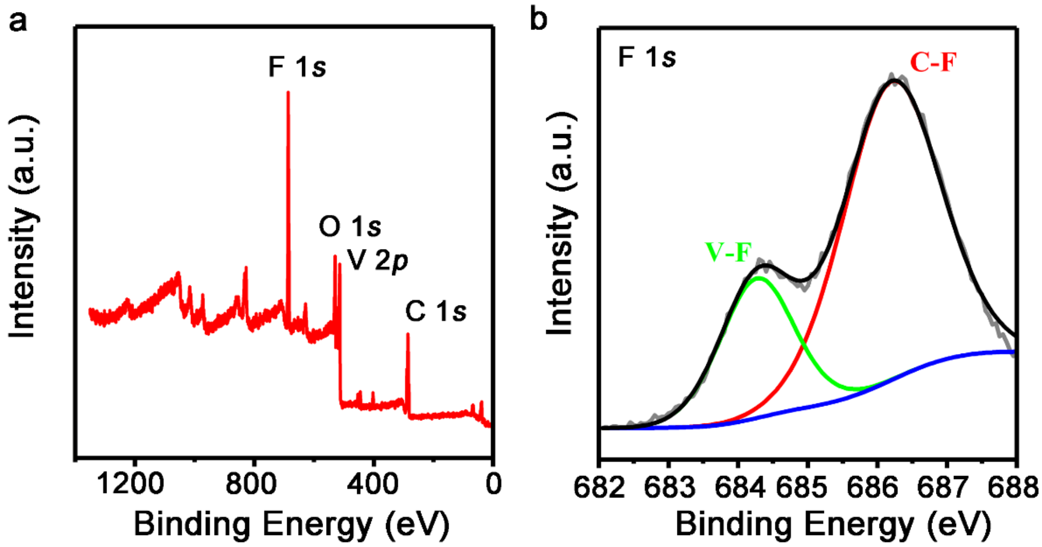

Supplementary Fig. 41 | XPS spectra of V_2C MXenzyme. (**a**) Survey spectrum of V_2C MXenzyme. (**b**) High-resolution XPS spectra of F 1*s*.

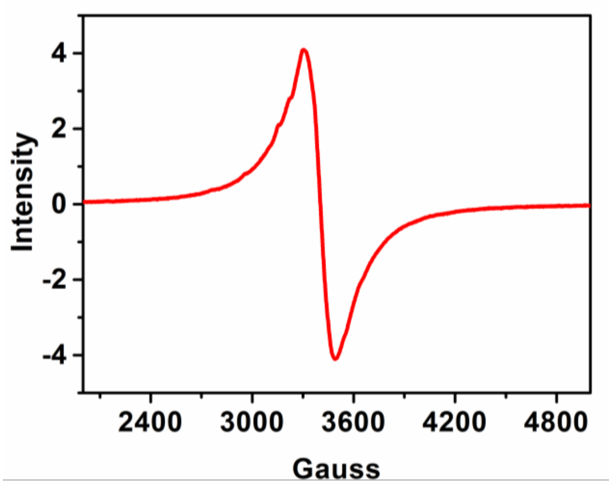

Supplementary Fig. 42 | ESR spectrum of the reaction mixture containing V_2C MXenzyme and H_2O_2.

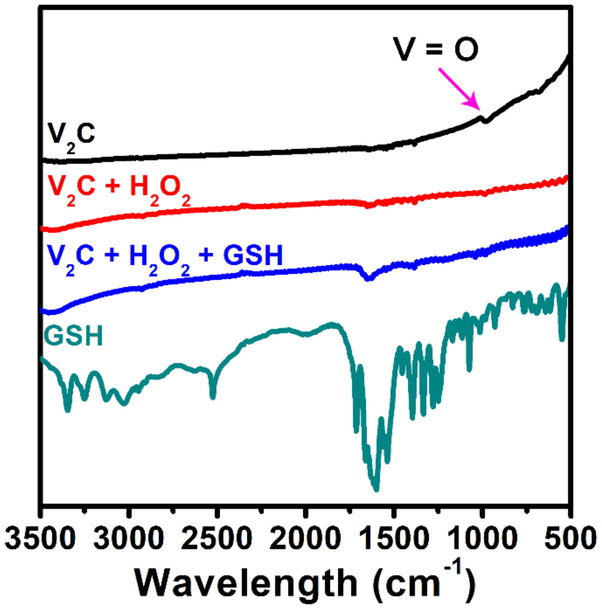

Supplementary Fig. 43 | FTIR spectra of the reaction mixture containing different components.

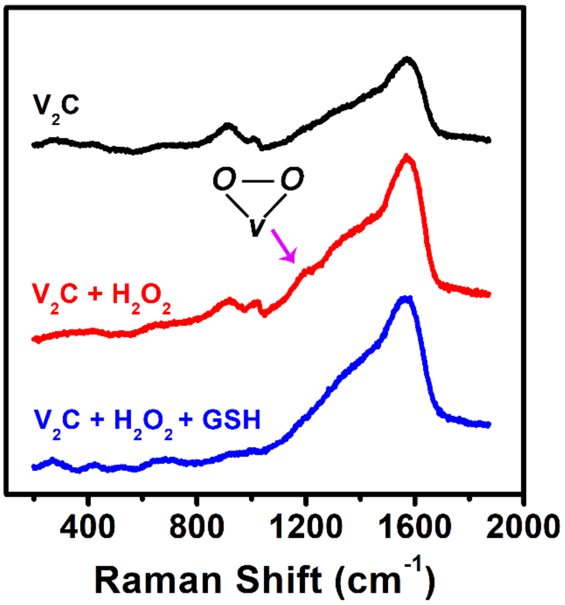

Supplementary Fig. 44 | Raman spectra of the reaction mixture containing different components.

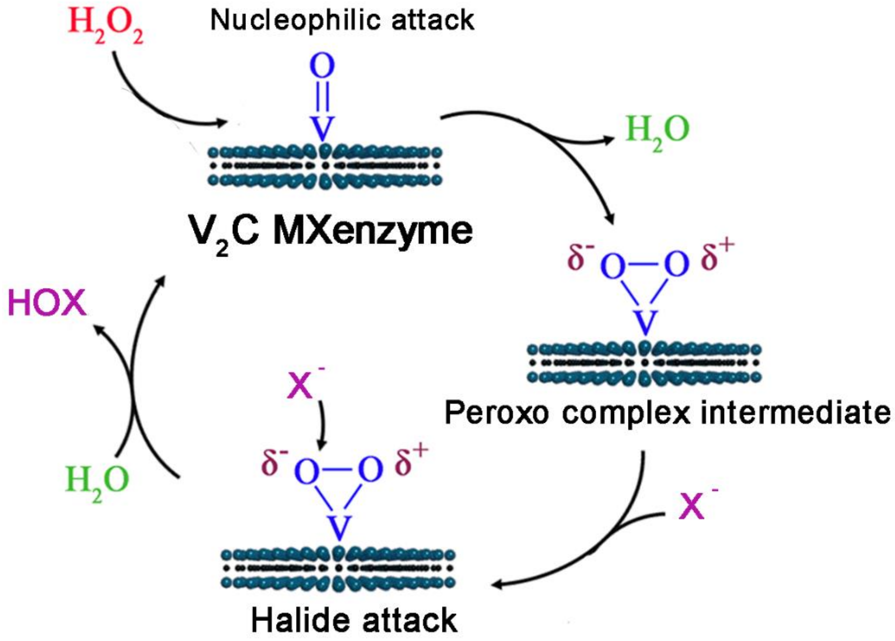

Supplementary Fig. 45 | Schematic illustration revealing the proposed mechanism of HPO-like activity of V_2C MXenzyme.

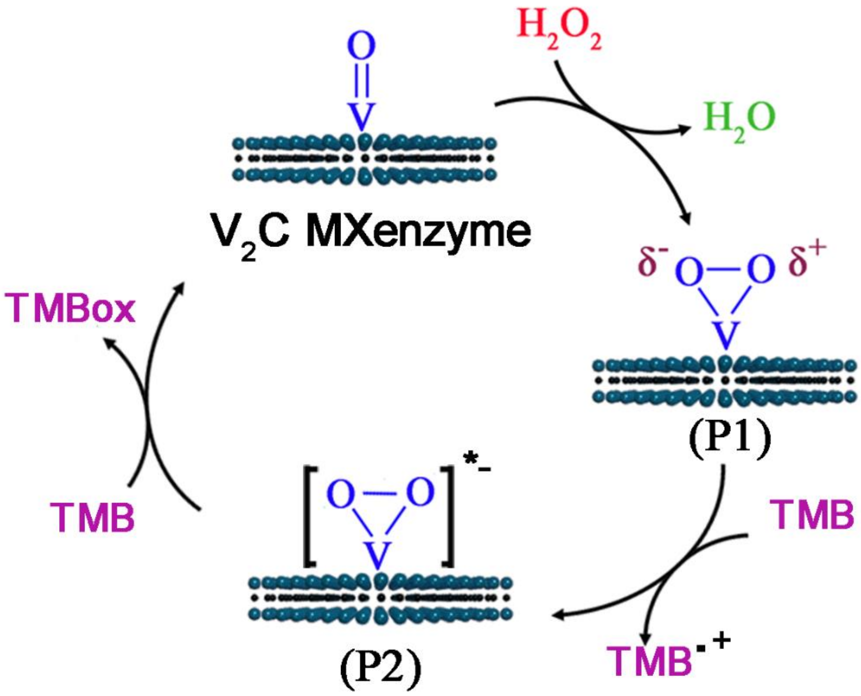

Supplementary Fig. 46 | Schematic illustration revealing the proposed mechanism of POD-like activity of V_2C MXenzyme.

Supplementary Discussion

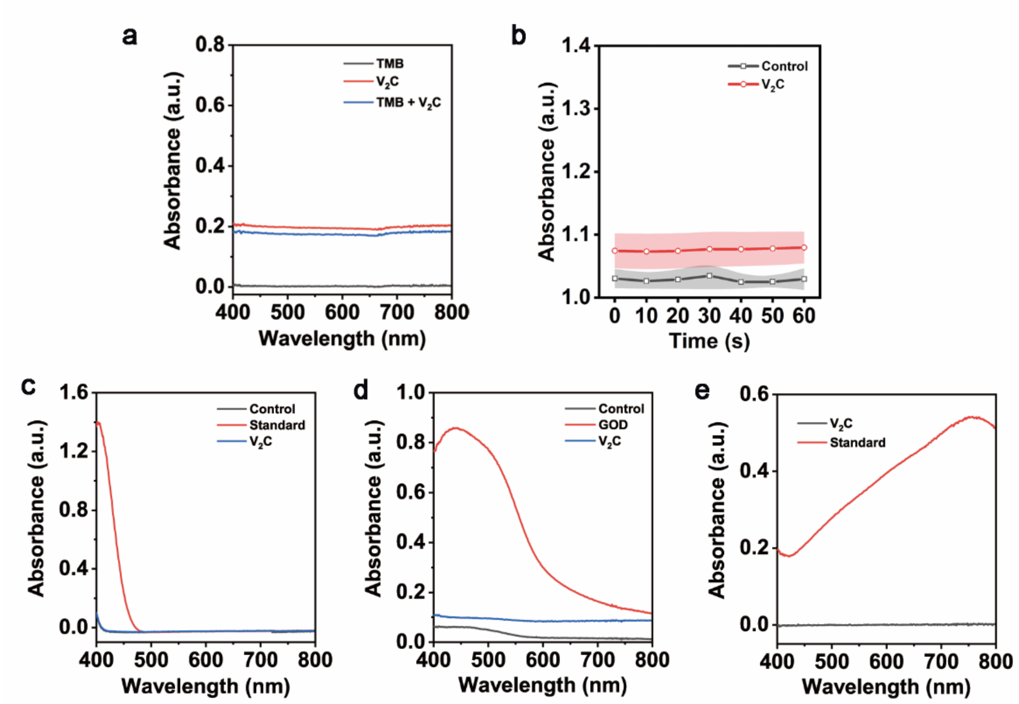

Supplementary Fig. 47 | Exploration of other enzyme-like activities. (**a**) OXD-like activity. (**b**) XOD-like activity (n = 3 for each group, data presented as Mean ± SD). (**c**) Phosphatase-like activity. (**d**) GOD-like activity. (**e**) Protease-like activity.

In addition, we further investigated the oxidase (OXD)-like activity, which reduces oxygen into water in the presence of a hydrogen (H) donor. In order to monitor the reaction, 3,3',5,5'-Tetramethylbenzidine (TMB) was introduced as the H donor, as the oxidized TMB develops a blue product with absorbance at 652 nm. V_2C MXene doesn't exhibit the capability to oxidize TMB in sodium acetate (NaAc) buffer (**Supplementary Fig. 47a**), revealing that it doesn't possess OXD-like activity. Furthermore, V_2C MXene cannot mimic xanthine oxidase (XOD) that catalyzes the sequential oxidation of hypoxanthine to xanthine and xanthine to uric acid and hydrogen peroxide (**Supplementary Fig. 47b**). Next, we assessed the phosphatase-like activity using *para*-nitrophenyl phosphate (pNPP), which becomes an intense yellow soluble product para-nitrophenol under alkaline conditions and can be conveniently at 405 nm on a spectrophotometer. As shown in **Supplementary Fig. 47c**, V_2C MXene doesn't catalyze the decomposition of pNPP to p-nitrophenol and phosphate, indicating no obvious phosphatase-like activity. Glucose oxidase (GOD)-like activity is determined by a coupled enzyme assay, in which GOD oxidizes D-glucose resulting in the production of hydrogen peroxide (H_2O_2) that reacts

with o-dianisidine, generating a colorimetric (500 nm) product, proportional to GOD present. Almost no significant absorbance of V_2C MXene with D-glucose is observed at 500 nm, confirming no GOD-like activity for V_2C MXene (**Supplementary Fig. 47d**). In addition, the protease can hydrolyze casein to produce tyrosine, which can reduce phosphomolybdic acid compound to tungsten blue, possessing a characteristic absorption peak at 680 nm. After investigation, we cannot find that V_2C MXene display protease-like activity (**Supplementary Fig. 47e**). Based on these results, V_2C MXene doesn't have OXD-, XOD-, phosphatase-, GOD- and protease-like activity. It is noted that there are thousands of enzymes in the nature and human body, such as lipases, amylase, maltase, trypsin, lactase, acetylcholinesterase, helicase, DNA polymerase and so on. Therefore, we cannot test all of them in this study. In future, we will launch a systematic study of the activities of V_2C MXene-based artificial enzymes.

Supplementary Data

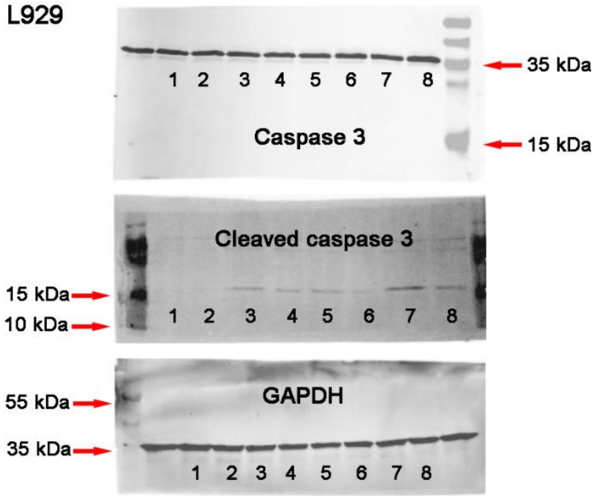

Supplementary Fig. 48 | Western blot data for Figure 7b. Caspase-3, cleaved caspase-3 and GAPDH expressions in L929 cells after different treatments (1. Control, 2. V_2C, 3. UV irradiation, 4. V_2C + UV irradiation, 5. H_2O_2, 6. V_2C + H_2O_2, 7. H_2O_2 + $FeSO_4$ and 8. V_2C + H_2O_2 + $FeSO_4$). Caspase 3 and GAPDH run on different gels under same conditions due to the molecular weight of Caspase 3 is close to that of GAPDH.

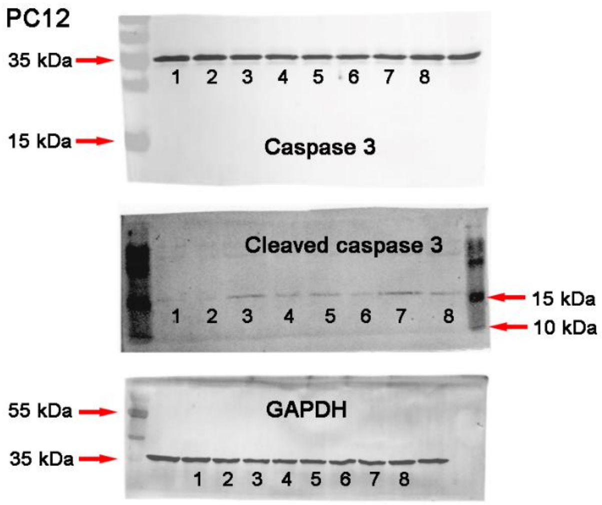

Supplementary Fig. 49 | Western blot data for Figure 7b. Caspase-3, cleaved caspase-3 and GAPDH expressions in PC12 cells after different treatments. **(**1. Control, 2. V_2C, 3. UV irradiation, 4. V_2C + UV irradiation, 5. H_2O_2, 6. $V_2C + H_2O_2$, 7. $H_2O_2 + FeSO_4$ and 8. $V_2C + H_2O_2 + FeSO_4$). Caspase 3 and GAPDH run on different gels under same conditions due to the molecular weight of Caspase 3 is close to that of GAPDH.
