# Supplementary material for: 2D vanadium carbide MXenzyme to alleviate ROS-mediated inflammatory and neurodegenerative diseases
Source: Nat Commun. 2021 Apr 13;12:2203. doi: 10.1038/s41467-021-22278-x (PMC8044242; doi:10.1038/s41467-021-22278-x)
Supplement: Supplementary file 3 — Reporting Summary [file 41467_2021_22278_MOESM3_ESM.pdf]

Corresponding Author Name: Yuehua Li and Yu Chen

Manuscript Number: NCOMMS-20-04230B

Reporting Checklist For Life Sciences Articles

This checklist is used to ensure good reporting standards and to improve the reproducibility of published results. For more information, please read Reporting Life Sciences Research. List items are standard for all Nature journal articles but may not apply to all disciplines or manuscripts.

Figure legends

☑ Check here to confirm that the following information is available in all relevant figure legends (or Methods section if too long):

- the **exact sample size (*n*)** for each experimental group/condition, given as a number, not a range;
- a **description of the sample collection** allowing the reader to understand whether the samples represent **technical or biological replicates** (including how many animals, litters, culture, etc.);
- a **statement of how many times the experiment shown was replicated in the laboratory**;
- **definitions of statistical methods and measures**: (For small sample sizes (n<5) descriptive statistics are not appropriate, instead plot individual data points)
  - o very common tests, such as *t*-test, simple χ^2 tests, Wilcoxon and Mann-Whitney tests, can be unambiguously identified by name only, but more complex techniques should be described in the methods section;
  - o are tests one-sided or two-sided?
  - o are there adjustments for multiple comparisons?
  - o **statistical test results**, e.g., ***P* values**;
  - o definition of '**center values**' as **median** or **mean**;
  - o definition of **error bars** as **s.d.** or **s.e.m.** or **c.i.**

This checklist will not be published. Please ensure that the answers to the following questions are reported in the manuscript itself. We encourage you to include a specific subsection in the Methods section for statistics, reagents and animal models. Below, provide the page number or section and paragraph number (e.g. "Page 5" or "Methods, 'reagents' subsection, paragraph 2").

Statistics and general methods	Reported in section/paragraph or page #:
1. How was the sample size chosen to ensure adequate power to detect a pre-specified effect size? (Give section/paragraph or page #)	Sample sizes were chosen based on the minimum number of [illegible]
For animal studies, include a statement about sample size estimate even if no statistical methods were used.	For animal studies, sample sizes were chosen based on the [illegible]
2. Describe inclusion/exclusion criteria if samples or animals were excluded from the analysis. Were the criteria pre-established? (Give section/paragraph or page #)	No animals were excluded from the analysis.
3. If a method of randomization was used to determine how samples/ animals were allocated to experimental groups and processed, describe it. (Give section/paragraph or page #)	The samples/animals were allocated to experimental groups and processed randomly.
For animal studies, include a statement about randomization even if no randomization was used.	The animals were randomly divided into different groups. [illegible]
4. If the investigator was blinded to the group allocation during the experiment and/or when assessing the outcome, state the extent of blinding. (Give section/paragraph or page #)	The investigator carried out the data collection and/or analysis without knowing the group assignment for each [illegible]
For animal studies, include a statement about blinding even if no blinding was done.	All these experiments were carried out in single-blinded [illegible]
5. For every figure, are statistical tests justified as appropriate?	Yes
Do the data meet the assumptions of the tests (e.g., normal distribution)?	Yes
Is there an estimate of variation within each group of data?	No
Is the variance similar between the groups that are being statistically compared? (Give section/paragraph or page #)	No

(Continues on following page)

Reagents — Reported in section/paragraph or page #:

6. To show that antibodies were profiled for use in the system under study (assay and species), provide a citation, catalog number and/or clone number, supplementary information or reference to an antibody validation profile (e.g., Antibodypedia, 1DegreeBio).

   Anti-tyrosine hydroxylase antibody (abcam, #ab112, rabbit polyclonal to tyrosine hydroxylase - neuronal marker, suitable for IHC-P/WB, reacts with rat), anti-4 Hydroxynonenal antibody (abcam, #ab46545, rabbit

7. Cell line identity:

   a. Are any cell lines used in this paper listed in the database of commonly misidentified cell lines maintained by ICLAC (also available in NCBI Biosample)?

   L929 and PC12 cells are not listed in the database of commonly misidentified cell lines.

   b. If yes, include in the Methods section a scientific justification of their use – indicate here on which page (or section and paragraph) the justification can be found.

   c. For each cell line, include in the Methods section a statement that specifies:

   - the source of the cell lines

   Institute of Biochemistry and Cell Biology, Chinese Academy of Sciences

   - have the cell lines been authenticated? If so, by which method?

   The cell line we used was morphologically confirmed according to the information provided by Shanghai Institutes for Biological Sciences, Chinese Academy of Sciences.

   - have the cell lines been tested for mycoplasma contamination?

   In this checklist, indicate on which page (or section and paragraph) the information can be found.

   The L929 and PC12 cell lines are free of mycoplasma contamination.

Animal Models — Reported in section/paragraph or page #:

8. Report species, strain, sex and age of animals

   Kunming mice (female, 6 weeks) and C57BL/6 mice (female, 6 weeks) were purchased from Slac Laboratory Animal Co., Ltd. (Shanghai, China). Page 25-27, "Methods" Section, "In vivo toxicity assay, ear inflammation models construction and treatment, ankle inflammation models construction and

9. For experiments involving live vertebrates, include a statement of compliance with ethical regulations and identify the committee(s) approving the experiments.

   All animal procedures were carried out in strict compliance with the guidelines of the Regional Ethics Committee for Animal Experiments approved by the Administrative

10. We recommend consulting the ARRIVE guidelines (PLoS Biol. 8(6), e1000412,2010) to ensure that other relevant aspects of animal studies are adequately reported.

Human Subjects — Reported in section/paragraph or page #:

11. Identify the committee(s) approving the study protocol.

    No related data in this manuscript.

12. Include a statement confirming that informed consent was obtained from all subjects.

    No related data in this manuscript.

13. For publication of patient photos, include a statement confirming that consent to publish was obtained.

    No related data in this manuscript.

14. Report the clinical trial registration number (at ClinicalTrials.gov or equivalent).

    No related data in this manuscript.

15. For phase II and III randomized controlled trials, please refer to the CONSORT statement and submit the CONSORT checklist with your submission.

    No related data in this manuscript.

16. For tumor marker prognostic studies, we recommend that you follow the REMARK reporting guidelines.

    No related data in this manuscript.

(Continues on following page)

Data Availability

Reported in section/paragraph or page #

17. Please provide a Data Availability statement in the Methods section under "Data Availability". Data availability statements should include, where applicable, accession codes, other unique identifiers and associated web links for publicly available datasets, and any conditions for access of non-publicly available datasets. Where figure source data are provided, statements confirming this should be included in data availability statements. Please refer our data availability and data citations policy for detailed guidance on information that must be provided in this statement.

The authors declare that all data needed to support the finding of this study are presented in the article and the Supplementary Information. Any other data related to this work are available from the corresponding authors upon reasonable request. A reporting summary for this article is available as a Supplementary Information file. Page 25, "Data availability" Section.

Data deposition in a public repository is mandatory for:

a. Protein, DNA and RNA sequences
b. Macromolecular structures
c. Crystallographic data for small molecules
d. Microarray data

Deposition is strongly recommended for many other datasets for which structured public repositories exist; more details on our data policy are available here. We encourage the provision of other source data in supplementary information or in unstructured repositories such as Figshare and Dryad. We encourage publication of Data Descriptors (see Scientific Data) to maximize data reuse

18. If computer code was used to generate results that are central to the paper's conclusions, include a statement in the Methods section under "**Code availability**" to indicate whether and how the code can be accessed. Include version information as necessary and any restrictions on availability.

No related data in this manuscript.
